# Methylotrophic methanogens and bacteria synergistically demethylate dimethylarsenate in paddy soil and alleviate rice straighthead disease

**DOI:** 10.1038/s41396-023-01498-7

**Published:** 2023-08-21

**Authors:** Chuan Chen, Lingyan Li, Yanfen Wang, Xiuzhu Dong, Fang-Jie Zhao

**Affiliations:** 1 State Key Laboratory of Crop Genetics and Germplasm Enhancement and Utilization, Jiangsu Provincial Key Laboratory for Organic Solid Waste Utilization, College of Resources and Environmental Sciences, Nanjing Agricultural University, Nanjing, 210095 China; 2 College of Life Sciences, University of Chinese Academy of Sciences, No.19(A) Yuquan Road, Shijingshan District, 100049 Beijing, China; 3 State Key Laboratory of Microbial Resources, Institute of Microbiology, Chinese Academy of Sciences, 100101 Beijing, China; 4 College of Resources and Environment, University of Chinese Academy of Sciences, No.19(A) Yuquan Road, Shijingshan District, 100049 Beijing, China

**Keywords:** Environmental microbiology, Biogeochemistry

## Abstract

Microorganisms play a key role in arsenic (As) biogeochemistry, transforming As species between inorganic and organic forms and different oxidation states. Microbial As methylation is enhanced in anoxic paddy soil, producing primarily dimethylarsenic (DMAs), which can cause rice straighthead disease and large yield losses. DMAs can also be demethylated in paddy soil, but the microorganisms driving this process remain unclear. In this study, we showed that the enrichment culture of methylotrophic methanogens from paddy soil demethylated pentavalent DMAs(V) efficiently. DMAs(V) was reduced to DMAs(III) before demethylation. 16S rRNA gene diversity and metagenomic analysis showed that *Methanomassiliicoccus* dominated in the enrichment culture, with *Methanosarcina* and *Methanoculleus* also being present. We isolated *Methanomassiliicoccus luminyensis* CZDD1 and *Methanosarcina mazei* CZ1 from the enrichment culture; the former could partially demethylate trivalent DMAs(III) but not DMAs(V) and the latter could demethylate neither. Addition of strain CZDD1 to the enrichment culture greatly accelerated DMAs(V) demethylation. Demethylation of DMAs(V) in the enrichment culture was suppressed by ampicillin, suggesting the involvement of bacteria. We isolated three anaerobic bacterial strains including *Clostridium* from the enrichment culture, which could produce hydrogen and reduce DMAs(V) to DMAs(III). Furthermore, augmentation of the *Methanomassiliicoccus*-*Clostridium* coculture to a paddy soil decreased DMAs accumulation by rice and alleviated straighthead disease. The results reveal a synergistic relationship whereby anaerobic bacteria reduce DMAs(V) to DMAs(III) for demethylation by *Methanomassiliicoccus* and also produce hydrogen to promote the growth of *Methanomassiliicoccus*; enhancing their populations in paddy soil can help alleviate rice straighthead disease.

## Introduction

Arsenic (As) is a ubiquitous contaminant in the environment. Arsenic exists in the environment in various chemical species differing in the toxicity to organisms [1]. Microorganisms play a key role in the transformation of As species, such as reduction and oxidation between the pentavalent [As(V)] and trivalent [As(III)] oxidation states, and methylation and demethylation of As compounds [1]. Arsenic methylation is catalyzed by As(III) *S*-adenosylmethionine methyltransferases (ArsMs) in microorganisms and AS3MT in higher eukaryotes [1–4], where arsenite is methylated stepwise to produce mono-, di- and tri-methylated As compounds. Arsenic methylation is enhanced under anoxic conditions, such as submerged paddy soils, likely because the substrate arsenite becomes more available and the ability to methylate arsenite is more widespread in anaerobes, such as sulfate-reducing bacteria and fermentative bacteria [5–8]. In paddy soils and rice grain, dimethylarsenic (DMAs) is the most prevalent methylated As species [5, 9–11]. Arsenic in DMAs and MMAs can be present in either the pentavalent [DMAs(V) and MMAs(V)] or trivalent [DMAs(III) and MMAs(III)] oxidation state, with the trivalent form being much more toxic than the pentavalent form [12, 13]. Both DMAs(III) and MMAs(III) have been found in anoxic paddy soil and rice plants [14–16], although both, especially the former, are unstable when exposed to air [17]. Excessive accumulation of DMAs in rice plants can cause a physiological disorder called straighthead disease, resulting in spikelet sterility and large yield losses [18–21]. The toxicity may be attributed to DMAs(III) rather than DMAs(V) [14]. The straighthead disease is prevalent in many rice growing regions, especially after upland soil is converted to paddy and in the upland–paddy rotation system [22, 23]; the reasons for this prevalence are unknown, but differences in microbial community are likely involved.

Opposite to the process of As methylation, methylated As compounds can be demethylated by some microorganisms. It has been shown that MMAs(III) is demethylated by the dioxygenase C−As lyase ArsI in some bacteria [24, 25]. DMAs can also be demethylated, although the mechanism remains unclear. Typically, DMAs accumulates in paddy soil initially after flooding of the soil, followed by gradual disappearance due to demethylation [5, 6, 26]. Thus, the concentration of DMAs in paddy soil and the level of DMAs accumulated in rice plants depend on not only As methylation but also demethylation of DMAs. Based on the experiments of inhibition of methanogenesis and ^13^C-labeled DMAs, we have previously shown that some methanogens likely mediate demethylation of DMAs in anoxic paddy soil [6]. However, the methanogens that can demethylate DMAs have not been identified. This knowledge could be highly useful for developing strategies to prevent or alleviate rice straighthead disease.

The objective of the present study was to identify methanogens that can demethylate DMAs in paddy soil. We showed that the H_2_-dependent methylotrophic methanogen *Methanomassiliicoccus* can demethylate DMAs(III) but not DMAs(V). We also found that anaerobic bacteria reduce DMAs(V) to DMAs(III) for the demethylation by *Methanomassiliicoccus* and also provide H_2_ for the growth of *Methanomassiliicoccus*. We further showed that augmentation of the *Methanomassiliicoccus-Clostridium* coculture decreased DMAs accumulation in rice plants and alleviated straighthead disease.

## Materials and methods

### Enrichment cultures of methanogens

Methanogenic enrichment cultures were constructed by inoculating 0.5 g of an arsenic contaminated (total As 86 mg kg^−1^) paddy soil collected from Chenzhou (CZ), Hunan province in China into 5 ml pre-reduced medium (Table S1) and incubated for 30 days at 30 °C. Three types of methanogenic substrates were used, including H_2_/CO_2_ (80%/20%, 0.1 MPa), 20 mM acetate and 20 mM methanol. A negative control without methanogenic substrates was included. DMAs(V) (80 μM) was added to all enrichment cultures. The methanol enrichment culture was transferred for 14 generations using the previous generation as inoculant (10% V:V). Methanol (20 mM) and DMAs(V) (20 μM) were added to each generation. In some experiments, DMAs(III) (20 μM) was used instead of DMAs(V). DMAs(III) was synthesized from DMAs(V) according to the procedure described elsewhere [27]. In one experiment, 2-bromoethanesulfonic acid (BES, 10 mM) and ampicillin (100 mg l^−1^) were added separately to the methanol enrichment culture to inhibit methanogens and bacteria, respectively. At different time points, enrichment cultures were sampled for determination of As species, the compositions of bacteria and archaea, and the *mtaB* gene abundance. Methane and hydrogen (H_2_) in the headspace were quantified using gas chromatograph (Agilent 8860 GC system, USA). The mass balance of As species was determined in the first-generation methanol enrichment culture at the end of the experiment by measuring the As species in the solution and sorbed by the soil residues, as well as volatile As in the headspace. Volatile As was trapped using chemotraps with silica gels impregnated with 10% (w/v) AgNO_3_ and quantified as described previously [7]. Arsenic species in the soil residues were extracted with 50 mM NH_4_H_2_PO_4_ and determined as described below.

### Design of specific primers for *mtaB* gene of *Methanomassiliicoccus*

MtaB (methanol: 5-hydroxy-benzimidazolyl-cobamide methyltransferase) could be used for phylogenetic analysis, because the MtaB sequences of *Methanomassiliicoccus* are not intermixed with members of Methanosarcinales and form robustly supported monophyletic groups [28]. Sixteen *mtaB* gene sequences of *Methanomassiliicoccus* and three of *Methanosarcina* were downloaded from GeneBank in NCBI and from assembled contigs of metagenomic sequencing of enrichment cultures in the present study. Multiple sequence alignments of all sequences were performed using the CLUSTALW program. Sequences of *mtaB* were conserved among the *Methanomassiliicoccus* group, but distinct from *mtaB* in *Methanosarcina* (Fig. S1A). Therefore, we designed a pair of primers (P3/P4) specifically targeting the *Methanomassiliicoccus mtaB* genes (Table S2). The specificity of the primers was verified using the genomic DNA of *Methanomassiliicoccus luminyensis* CZDD1, *Methanomassiliicoccus luminyensis* B10 and *Methanosarcina mazei* CZ1 as templates. Using the P3/P4 primers, *mtaB* in *Methanomassiliicoccus luminyensis* CZDD1 and B10 was successfully amplified, but not from *Methanosarcina mazei* CZ1 (Fig. S1B). A single PCR product as indicated by agarose gel electrophoresis was amplified from both paddy soil and enrichment culture (Fig. S1C). The primer specificity was further verified on a clone library of *mtaB* gene amplified from paddy soil. Among the 70 clones sequenced, 94% were identified by BLAST on the NCBI (https://blast.ncbi.nlm.nih.gov/) to be *Methanomassiliicoccus* and the remainder was unclassified (Fig. S1D).

### DNA extraction and quantitative real-time PCR

Total DNA in different generations of enrichment cultures were extracted using a Power Soil DNA isolation Kit (QIAGEN, Germany). DNA concentration was determined by using a NanoDrop 2000C spectrophotometer (Thermo Scientific, Wilmington, USA). The absolute abundances of *mtaB* genes were quantified using the primers P3/P4 (Table S2) and conducted with a real-time PCR detection system (Bio-Rad CFX96, USA). Bacterial 16S rRNA gene abundance was quantified using the primers P5/P6 (Table S2).

### High-throughput sequencing and metagenomic assembly

Bacterial and archaeal community structures in enrichment cultures were analyzed by using high-throughput sequence based on bacterial and archaeal 16S rRNA genes. The V4–V5 regions of bacterial and archaeal 16S rRNA were amplified using the primers P5/P6 and P7/P8, respectively (Table S2). The amplicons were purified, quantified and sequenced on a Mieseq platform (Illumina; PE250 mode) (Shanghai Biozeron Biotech. Co., LTD). Raw sequences were quality filtered and assembled. The sequences were processed using Quantitative Insights Into Microbial Ecology (QIIME, V1.9.0 http://qiime.org/scripts/assign_taxonomy.html). Effective sequences were grouped into operational taxonomic units (OTUs) based on similarity score of 97% using UPARSE (version 7.1 http://drive5.com/uparse/). Representative sequences were selected and the taxonomic classification of representative sequences for each OTU were identified by uclust algorithm (http://www.drive5.com/usearch/manual/uclust_algo.html) against SILVA(SSU138.1) 16S rRNA database based on the confidence threshold of 80% [29]. Total genomic DNA in sixth generation of enrichment cultures was extracted and used for metagenomic analysis. The quality of DNA was determined using Qubit (Thermo Fisher Scientific, Waltham, MA) and Nanodrop (Thermo Fisher Scientific, Waltham, MA). DNA libraries were constructed and sequenced on the Illumina Novaseq 6000 sequencer using the pair-end technology (PE 150). The raw sequencing reads of three enriched samples were processed with MetaWRAP v1.2.2, and the integrated pipeline for metagenomic data analysis. Read_qc module in meta-WRAP was used to filter raw paired-end reads. Clean reads were assembled individually using MEGAHIT v1.1.3 with default parameters, and short contigs (<1000 bp) were removed. The initial metagenome-assembled genomes (MAGs) were recovered according to the Multiple contig binning methods [CONCOCT v1.0.0, MetaBAT2 v2.12.1 [30] and MaxBin2 v2.2.6 [31]] applied in binning module of MetaWRAP. Final MAGs were obtained after three replicate MAG sets were merged and refined using the bin_refinement module in metaWRAP. Taxonomy prediction of the selected MAGs was classified using GTDB-Tk v1.0.2 [32] (classify_wf workflow, default parameter). Functional annotation was predicted using Prokka v1.13 [33] and KofamKOALA [34].

### Isolation and identification of methylotrophic methanogens and bacteria

Single strains of methanogens and bacteria were isolated by using the Hungate rolling tube technique [35]. Briefly, the methanol enrichment culture (the 6th generation) was diluted in a 10-fold series from 10^−1^ to 10^−7^. Each 500 μl dilution was injected into anaerobic tubes containing 5 ml of agar medium and incubated for 30 days, with or without H_2_ supplementation. Methanol (20 mM), H_2_ (±0.1 MPa), and ampicillin (100 mg l^−1^) were added into the tubes. After 30 days incubation, methanogenic colonies were picked into liquid medium in a glovebox filled with 100% N_2_. For isolation of bacteria, 20 mM methanol and 10 mM BES were applied. After culture for 2 weeks, bacterial colonies were picked into fresh liquid medium and purified to obtain pure cultures. The methanogenic and bacterial isolates were identified based on the similarity of 16S rRNA gene sequences, which were amplified using the primers P9/P10 and P11/P12, respectively (Table S2). Seventeen 16S rRNA sequences of methylotrophic methanogens from NCBI database closely related to the isolates obtained in the present study were selected as reference sequences and used for constructing the polygenic tree using neighbor-joining algorithm with software MEGA 11.0. The tree topology was estimated by bootstraps based on 1000 replications. Twenty reference sequences of bacteria closely related to the bacterial isolates obtained in the present study were downloaded, and a phylogenetic tree of bacterial isolates was also established as above.

### Demethylation of DMAs by the monocultures of *Methanomassiliicoccus luminyensis* CZDD1 and *Methanosarcina mazei* CZ1 or the coculture of *Methanomassiliicoccus-Clostridium*

Strains *Methanomassiliicoccus luminyensis* CZDD1 and *Methanosarcina mazei* CZ1 were isolated in the present study. Strain CZDD1 was cultured in a medium with exogenously added H_2_ (0.1 MPa), 20 mM methanol, ampicillin (100 mg l^−1^), DMAs(V) (5 μM) or DMAs(III) (0.5, 1 or 5 μM). Strain CZ1 was cultured in a medium containing 20 mM methanol with or without DMAs(V) (5 μM). In a separate experiment, the two strains were cultured to the mid-log phase and inoculated into the methanol enrichment culture (1st generation) with the addition of 20 μM DMAs(V). A control without inoculation was included. Each experiment was replicated three times. After culture for 36 days at 30 °C, CH_4_ in the headspace and As species in the culture medium were determinated.

Three anaerobic bacteria, *Clostridium malenominatum* CZB5, *Tissierella carlieri* CZB10 and *Clostridium subterminale* CZB11, were isolated from the methanol enrichment culture. These strains were cultured in a medium with 20 μM DMAs(V) (Table S1) at 30 °C. Syntrophic cocultures between methanogenic strain *Methanomassiliicoccus luminyensis* CZDD1 and bacterial strain *Clostridium malenominatum* CZB5, *Tissierella carlieri* CZB10 and *Clostridium subterminale* CZB11 were constructed. The bacterial strains were precultured to the mid-log phase. Methanogenic and bacterial strains were mixed in equal volume in a medium with 20 mM methanol, DMAs(V) (20 μM) or DMAs(III) (1 μM) and incubated at 30 °C. Methane and H_2_ in the headspace, and As species in the culture medium were determined. In addition, ^13^C-labeled DMAs(V) (5 μM) was synthesized according to Chen et al. [6]. ^13^C-DMAs(V) (5 µM) was added to the *Methanomassiliicoccus*-*Clostridium* coculture. ^13^C/^12^C isotope ratios of methane in the headspace were determined using GC - IRMS (Thermo Fisher Scientific, Germany).

### Soil incubation

A paddy soil was collected from Tancheng (TC), Shangdong province, China, where straighthead disease was observed in rice crops [19]. Twenty g soil (<2 mm) were placed into 100 ml serum bottles, to which 40 ml deionized water was added. The bottles were flushed with N_2_ for 30 min, sealed using rubber stoppers, secured with aluminum crimp caps and incubated at 30 °C in the darkness inside a glovebox (100% N_2_). Soil was incubated for 4 days when DMAs concentration in the soil solution reached a peak. Subsequently, 5 ml inoculants of *Methanomassiliicoccus luminyensis* CZDD1, *Clostridium malenominatum* CZB5, or the *Methanomassiliicoccus*-*Clostridium* coculture were added to the soil. A control without inoculant was included. Each treatment was replicated in three bottles. Inoculants were precultured to the mid-log phase before being introduced to the soil. Soil solution was collected at different time points during 15 days of incubation for the determination of As species [25].

### Pot experiment with rice

TC soil weighing 1.8 kg was placed into a 2 l plastic cup. Compound fertilizers (0.18 g, N: P_2_O_5_: K_2_O = 15: 15: 15) were mixed into the soil. The soil was flooded with a 3–5 cm layer of standing water above the soil surface for 1 week. One rice seedling (cultivar Jingliangyouhuazhan) was transplanted into each pot. Three treatments were established including control, the addition of *Clostridium malenominatum* CZB5 or the *Methanomassiliicoccus*-*Clostridium* coculture (150 ml kg^−1^ soil). Each treatment was replicated in three pots. The inoculants were injected into the soil at about 5 cm below the soil surface at the late tilling stage of rice growth. Rice was grown in a greenhouse with 16 h light (30 °C)/8 h dark (22 °C) photoperiod. After inoculation for 1, 7 and 30 days, soil was collected and total DNA was extracted. The abundances of *mtaB* and methyl-coenzyme reductase M coding gene (*mcrA*) were quantified. At rice maturity, the symptoms of straighthead disease and seed setting rate of rice were recorded. Arsenic species in the husks were extracted using 1% HNO_3_ and determined by using HPLC-ICP-MS [19].

### Demethylation of DMAs in different paddy soils

Six paddy soils and six upland soils (Table S3) were incubated under flooded conditions. Twenty grams of each soil were placed into a 100 ml serum bottle, to which 40 ml ultra-pure water was added. The bottles was purged with N_2_ for 30 min, sealed using rubber stoppers, secured with aluminum crimp caps and preincubated at 25 °C in the darkness. Each soil was replicated in 3 bottles. After incubation for 14 days, 40 μmol kg^−1^ DMAs(V) was added into each bottle inside an anaerobic glovebox. All soils were incubated for another 14 days. Before and after incubation, As species in solution and soil solid phase were determined. Arsenic species in the solid phase were extracted by using 50 mM NH_4_H_2_PO_4_ (M:V = 1:10) for 12 h and centrifuged [36]. Supernatant was collected and used for As species analysis. After incubation, a portion of soil was collected for DNA extraction. The absolute abundance of *mcrA* for total methanogens, *mtaB* specific for *Methanomassiliicoccus* were quantified by Real-Time PCR.

### Analysis of As species

Two methods were used for As species analysis. To test DMAs demethylation by enrichment cultures and isolates, As species in the culture medium were oxidized to their corresponding pentavalent forms using H_2_O_2_ (1 ml culture with 200 μl 30% H_2_O_2_), which stabilized the As species during sample preparation and quantification. Arsenic species were determined by using high performance liquid chromatography–inductively coupled plasma mass spectrometry (HPLC-ICP-MS, NexION 300X, PerkinElmer, USA), using an anion exchange column (Hamilton PRP 100) and a phosphate/nitrate mobile phase (8.5 mM NH_4_H_2_PO_4_ and 8.5 mM NH_4_NO_3_, pH 6.0) [19]. Arsenic species in rice husks from the pot experiment were extracted using 1% HNO_3_ and determined by HPLC-ICP-MS [19]. To determine DMAs(III) and MMAs(III), which are unstable in oxic conditions, As species in the culture medium were preserved with 8 mM diethyldithiocarbamic acid diethylammonium (DDDC) and quantified by HPLC-ICP-MS using a C18 column (Atlantis DC18, Waters) and a mobile phase containing 5 mM tetrabutylammonium hydroxide (TBAH), 3 mM malonic acid and 5% methanol [14, 37].

## Results

### Demethylation of DMAs(V) by enrichment cultures of methylotrophic methanogens

**Fig. 1:**
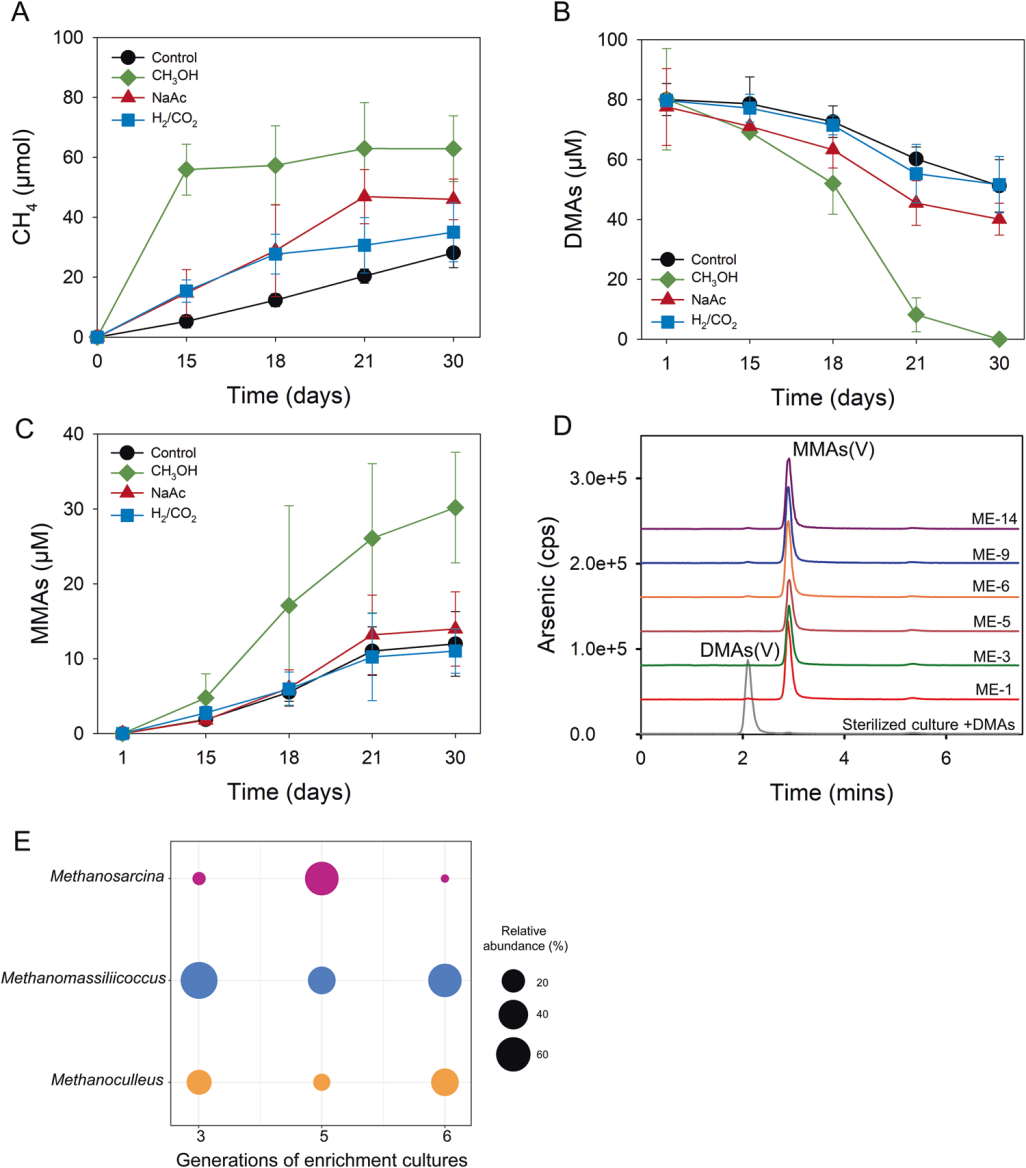
Demethylation of DMAs(V) by different methanogen enrichment cultures of a paddy soil. **A** Production of methane; **B** demethylation of DMAs(V); **C** production of MMAs of different methanogen enrichment cultures. **D** HPLC-ICP-MS chromatograms showing demethylation of DMAs(V) and production of MMAs(V) by different generations of methanol enrichment culture, after oxidation with H_2_O_2_. **E** The composition of methanogens in the 3rd, 5th and 6th generations of methanol enrichment cultures. Data in (**A**–**C**) are means ± SD (*n* = 3). Data in (**E**) are means of three replicates (*n* = 3).

To investigate which methanogenic pathway (hydrogenotrophic, acetoclastic and methylotrophic) is responsible for the demethylation of DMAs(V), we determined the demethylation activities of the methanogenic enrichment cultures from CZ paddy soil amended with either H_2_/CO_2_, acetate or methanol. During 30 days of culture, the enrichment culture amended with methanol produced the highest methane yield, followed by acetate and H_2_/CO_2_ (Fig. 1A). The control also produced some CH_4_, likely because the first generation of enrichment culture contained some soil residues that could be utilized by methanogens. DMAs(V) (80 μM) added to the methanol enrichment culture was completely demethylated (Fig. 1B). In contrast, the H_2_/CO_2_ enrichment culture did not enhance DMAs(V) demethylation compared with the control; both groups demethylated 28.5–35.2% of the added DMAs(V) after 30 days. In the acetate enrichment culture, 48.3% of the DMAs(V) added was demethylated. Concurrent with DMAs(V) demethylation was the production of MMAs(V); the amount of MMAs(V) in the solution accounted for 13.8–40.5% of the DMAs(V) demethylated (Fig. 1C and Table S4). The concentration of inorganic As (iAs), the final product of demethylation, also increased in the methanol enrichment culture (Fig. S2). Some of the iAs and MMAs(V) could be sorbed by the soil residue present in the enrichment cultures, which contained 0.5 g soil in 5 ml culture solution. We determined the mass balance in the methanol enrichment culture at the end of the experiment by measuring As species in the solution and the soil residues, as well as volatile As in the headspace (Table S4). Volatile As, MMAs(V) and iAs in both the solution and the solid phases accounted for 0.04%, 62% and 37%, respectively, of the DMAs(V) added initially, indicating a near complete recovery. These results suggest that the methylotrophic methanogens facilitate DMAs(V) demethylation, whereas the hydrogenotrophic methanogenic pathway does not contribute to DMAs(V) demethylation.

The methanol enrichment culture was successively subcultured for 14 generations. Each generation of the subculture retained the ability to produce methane (data not shown) and to demethylate DMAs(V) (Fig. 1D). Sequencing of archaeal 16S rRNA in the 3rd, 5th and 6th generations of subculture showed that two genera of methylotrophic methanogens, *Methanomassiliicoccus* and *Methanosarcina*, were enriched, with *Methanomassiliicoccus* being consistently the most abundant (20–72% of total archaea) in each generation of the enrichment and *Methanosarcina* exhibiting varied abundance (Fig. 1E). The hydrogenotrophic methanogen *Methanoculleus* was also present in the enrichment (7–33%).

### Isolation of methylotrophic methanogens

We performed metagenomic analysis of the 6th generation of the methanol enrichment culture and obtained 17 methanogenic MAGs, among which 8 belonged to Methanomassiliicoccales with 66.8–100% genome completeness and <2.4% contamination (Fig. S3). The other nine MAGs were clustered to hydrogenotrophic Methanomicrobiales, with >95.9% genome completeness and 0.21–4.76% contamination.

**Fig. 2:**
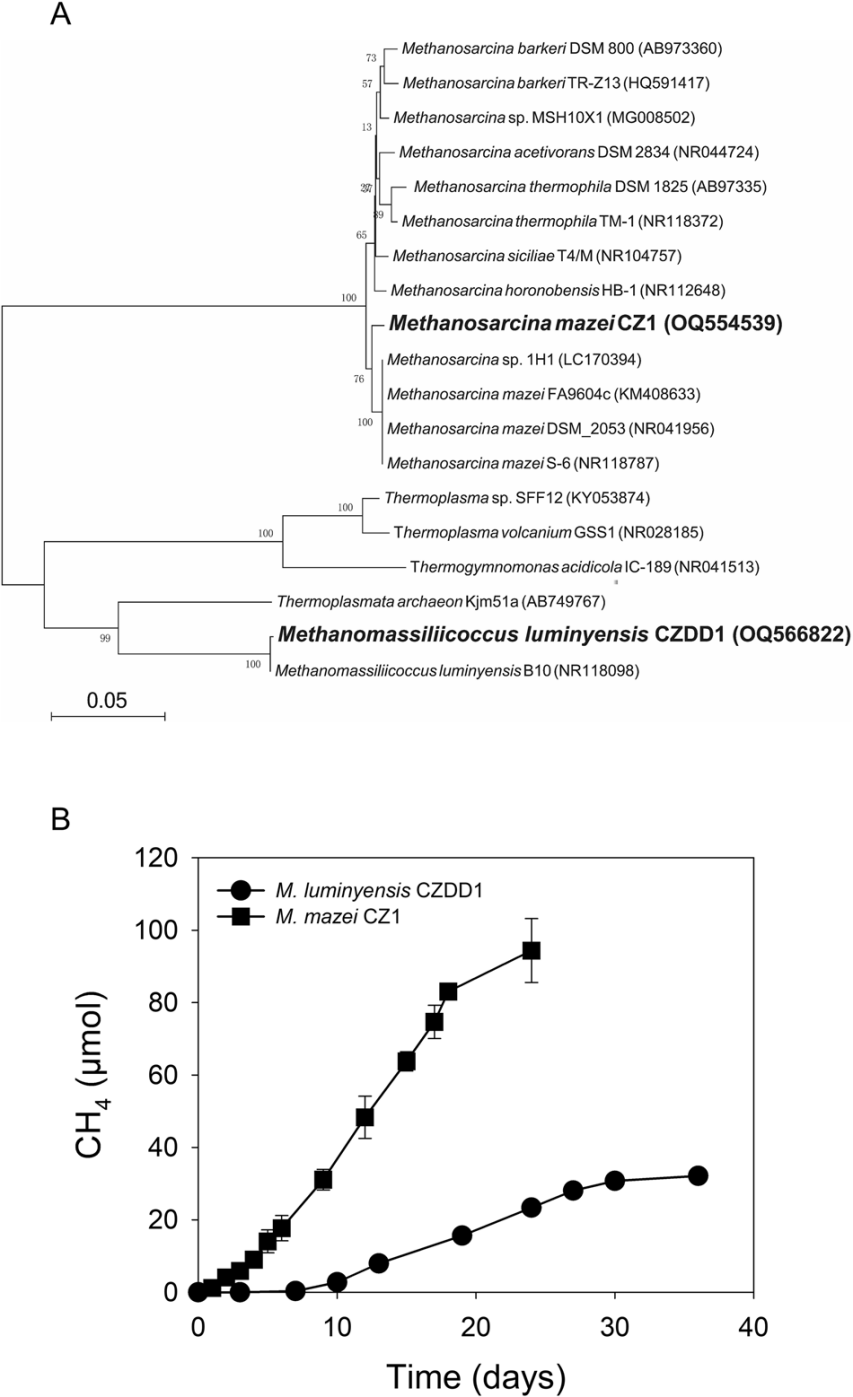
Isolation of two methylotrophic methanogens from the methanol enrichment culture of a paddy soil. **A** Phylogenetic relationship of *Methanomassiliicoccus luminyensis* CZDD1 and *Methanosarcina mazei* CZ1 with other known methylotrophic methanogens. **B** Methane production by strains *Methanomassiliicoccus luminyensis* CZDD1 and *Methanosarcina mazei* CZ1 cultured with 20 mM methanol and 20 μM DMAs(V), supplemented with H_2_ for strain CZDD1. Data in (**B**) are means ± SD (*n* = 3).

We isolated two methane-producing pure strains from the 6th generation of the methanol enrichment culture. Based on 16S rRNA gene homolog (Fig. 2A), strain CZ1 was identified as *Methanosarcina mazei* at 99.4% similarity with that of *Methanosarcina mazei* FA9604c, and strain CZDD1 as *Methanomassiliicoccus luminyensis* having 99.08% similarity with that of *Methanomassiliicoccus luminyensis* B10 isolated from human feces. We grew *Methanosarcina mazei* CZ1 in methanol and *Methanomassiliicoccus luminyensis* CZDD1 in methanol supplemented with H_2_ as the latter is a H_2_-dependent methylotrophic methanogen [38]. *Methanosarcina mazei* CZ1 produced more CH_4_ than *Methanomassiliicoccus luminyensis* CZDD1 (Fig. 2B).

### *Methanomassiliicoccus luminyensis* CZDD1 accelerated DMAs(V) demethylation in the methanol enrichment culture

We tested whether *Methanosarcina mazei* CZ1 or *Methanomassiliicoccus luminyensis* CZDD1 could demethylate DMAs(V) in pure culture with methanol (20 mM) and DMAs(V) (20 µM) as the substrates. H_2_ (0.1 MPa) was also provided for CZDD1. Over a period of 30 days, while methane was produced by both strains, no DMAs(V) was demethylated by either strain (Fig. S4).

**Fig. 3:**
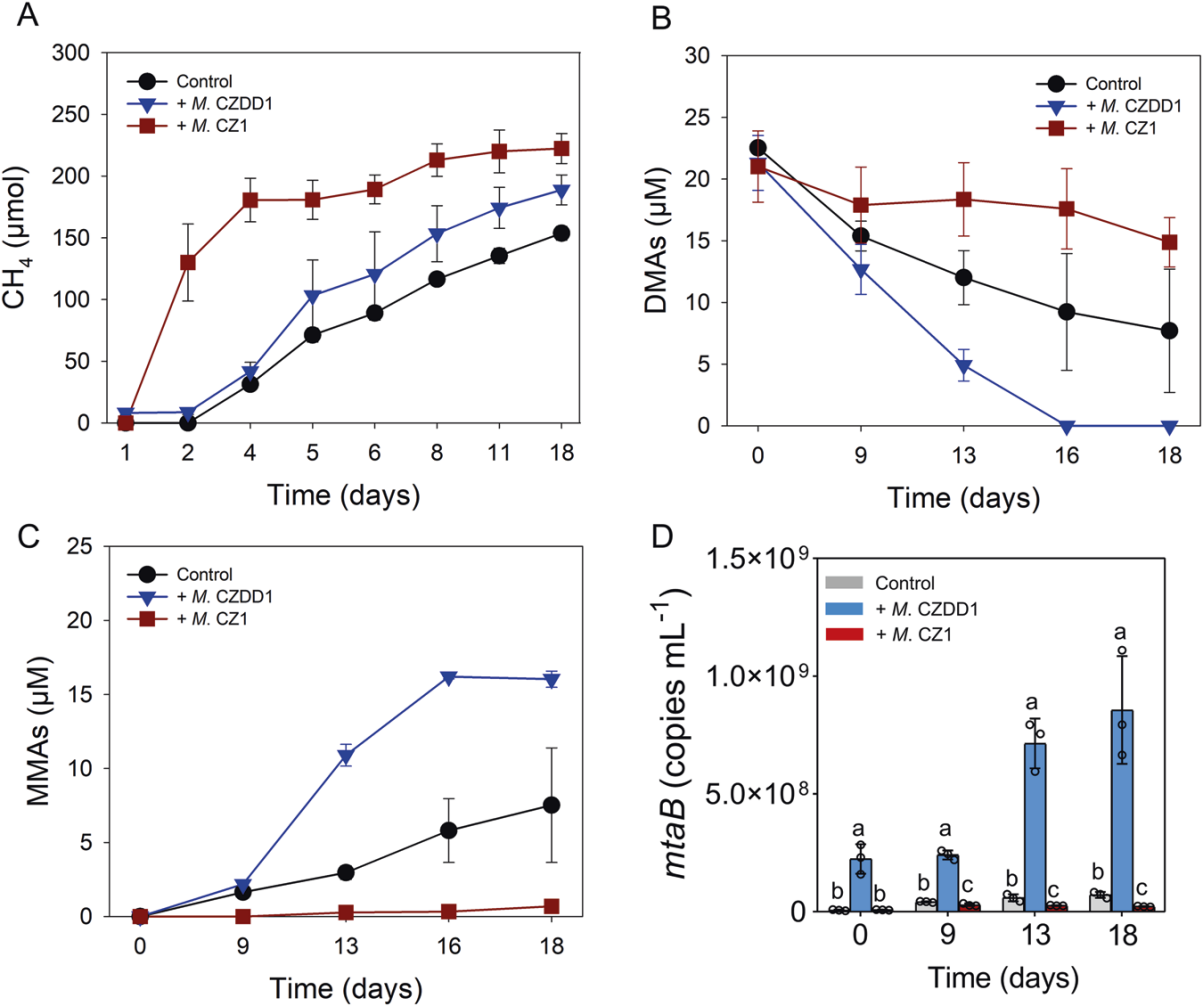
*Methanomassiliicoccus luminyensis* CZDD1 enhanced DMAs(V) demethylation by methanol enrichment culture. The effects of addition of *Methanomassiliicoccus luminyensis* CZDD1 or *Methanosarcina mazei* CZ1 to methanol enrichment culture on (**A**) methane production, (**B**) demethylation of DMAs(V), (**C**) production of MMAs(V), and (**D**) the absolute abundance of *Methanomassiliicoccus* specific *mtaB*. Data are means ± SD (*n* = 3). Different letters in (**D**) indicates significant difference at *p* < 0.05 between treatments (Tukey’s test).

The lack of DMAs(V) demethylation by the pure culture of *Methanosarcina mazei* CZ1 or *Methanomassiliicoccus luminyensis* CZDD1 could be because demethylation requires synergistic actions from other microorganisms. To test this hypothesis, we added pregrown culture of either strains to the 1st generation of the methanol enrichment culture. The addition of *Methanosarcina mazei* CZ1 greatly increased methane production compared with the control, whereas the addition of *Methanomassiliicoccus luminyensis* CZDD1 only marginally enhanced methane production (Fig. 3A). The addition of *Methanomassiliicoccus luminyensis* CZDD1 accelerated the demethylation of DMAs(V) and the production of MMAs(V) markedly compared with the control, whereas the addition of *Methanosarcina mazei* CZ1 suppressed DMAs(V) demethylation (Fig. 3B, C). Using the information of the genome sequence of *Methanomassiliicoccus luminyensis* CZDD1 and fifteen *mtaB* sequences of other strains and contigs belonging to *Methanomassiliicoccus*, we designed a specific primer pair targeting the *mtaB* gene for quantification (see Materials and Methods). The addition of *Methanomassiliicoccus luminyensis* CZDD1 increased the *mtaB* copy number by 5.7–32.2 fold compared with the control (Fig. 3D). In contrast, the addition of *Methanosarcina mazei* CZ1 decreased the *mtaB* copy number by 30.4–68.7% (*p* < 0.05). Analysis of archaeal 16S rRNA showed that the addition of *Methanomassiliicoccus luminyensis* CZDD1 significantly increased its relative abundance by 95%, but decreased the abundance of *Methanosarcina* and *Methanoculleus* (Fig. S5). These results suggest that *Methanomassiliicoccus luminyensis* CZDD1, but not *Methanosarcina mazei* CZ1, accelerated DMAs(V) demethylation. In fact, the addition of *Methanosarcina mazei* CZ1 appeared to suppress the growth of *Methanomassiliicoccus luminyensis* CZDD1, resulting in a suppression of DMAs(V) demethylation.

### Trivalent DMAs(III) is the substrate for demethylation

**Fig. 4:**
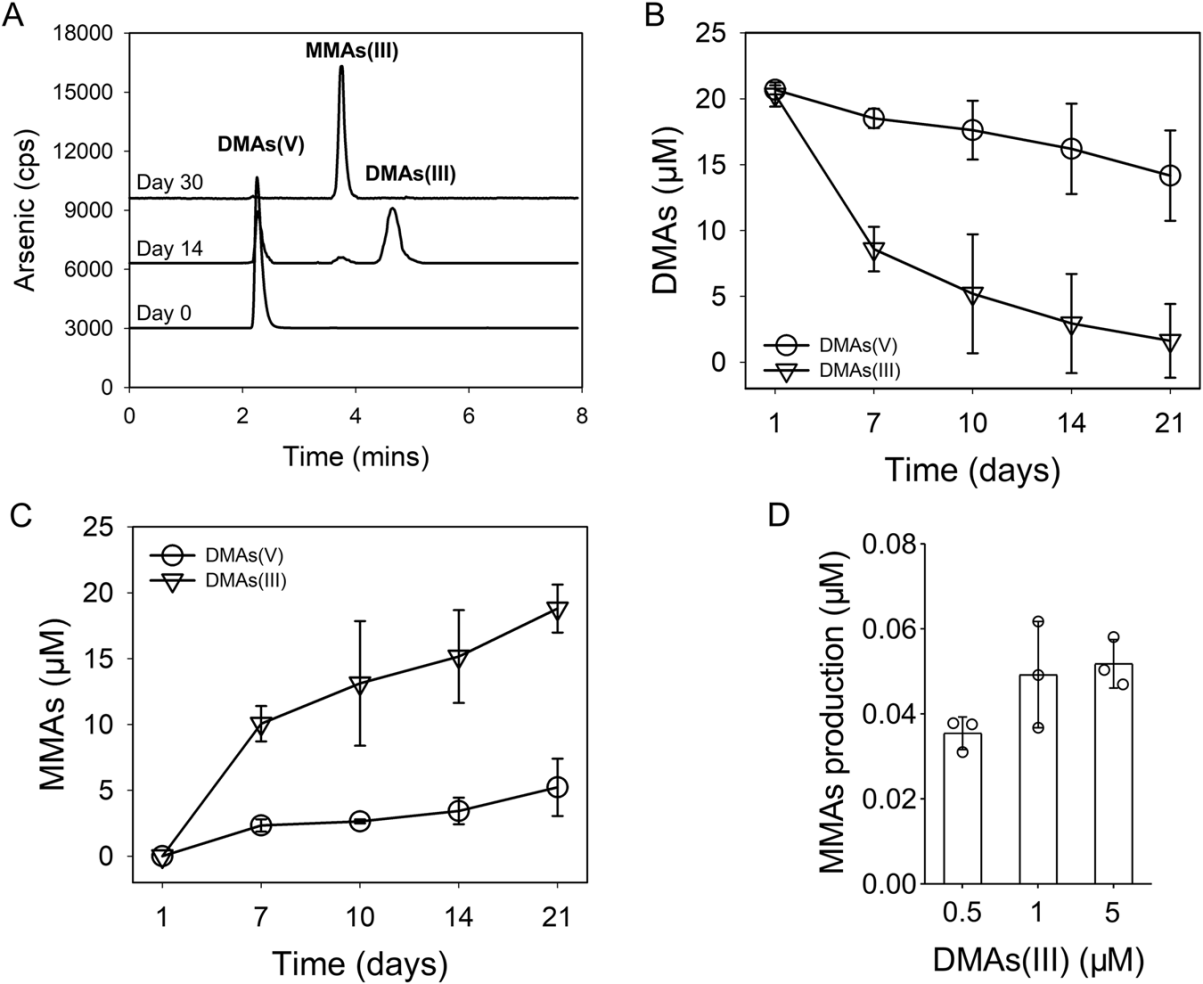
Demethylation of DMAs(III) and DMAs(V) by the methanol enrichment culture of a paddy soil. **A** HPLC-ICP-MS chromatograms showing As species in the methanol enrichment culture with 20 μM DMAs(V) during incubation. Trivalent As species were preserved with DDDC and separated by a C18 column before ICP-MS determination. **B** Demethylation rate of DMAs(V) and DMAs(III) by the methanol enrichment culture. **C** MMAs production in the methanol enrichment culture amended with DMAs(V) or DMAs(III). **D** Production of MMAs in the monoculture of *Methanomassiliicoccus luminyensis* CZDD1 supplemented with 0.5, 1 or 5 μM DMAs(III). Data in (**B**–**D**) are means ± SD (*n* = 3).

In the experiments described above, DMAs(V) was added to the enrichment cultures or pure cultures of methanogens. It is possible that DMAs(V) needs to be reduced to DMAs(III) before demethylation, as has been shown for bacterial demethylation of MMAs(V) [39]. We have recently shown that DMAs(III) is present in anoxic paddy soils and some anaerobes are able to reduce DMAs(V) to DMAs(III) [14]. To test whether DMAs(V) is reduced to DMAs(III) prior to demethylation, we determined As species transformation on day 0, 14 and 30 after the addition of DMAs(V) to the 14th generation of the methanol enrichment culture, using a protocol that preserves and quantify DMAs(III) [14, 37]. After 14 days, DMAs(V) was partially reduced to DMAs(III) and a trace amount of MMAs(III) was produced (Fig. 4A). After 30 days, both DMAs(V) and DMAs(III) had disappeared and MMAs(III) was produced quantitatively, suggesting that reduction of DMAs(V) to DMAs(III) precedes demethylation. In a further experiment, we found that DMAs(III) was demethylated much faster than DMAs(V) by the 6th generation of the methanol enrichment culture; by day 21, 92% of the added DMAs(III) was demethylated, compared with 31% of the added DMAs(V) (Fig. 4B, C). These results suggest that reduction of DMAs(V) to DMAs(III) may be the rate-limiting step for its demethylation.

We next tested whether pure cultures of *Methanomassiliicoccus luminyensis* CZDD1 and *Methanosarcina mazei* CZ1 could demethylate DMAs(III). After 30 days of culture with the addition of 0.5–5 μM DMAs(III), *Methanomassiliicoccus luminyensis* CZDD1 demethylated 1–7% of the added DMAs(III) to MMAs (Fig. 4D), indicating a weak ability to demethylate DMAs(III). In contrast, *Methanosarcina mazei* CZ1 could not demethylate DMAs(III) (data not shown).

### The presence of bacteria enhances demethylation of DMAs(V) by methanogens

**Fig. 5:**
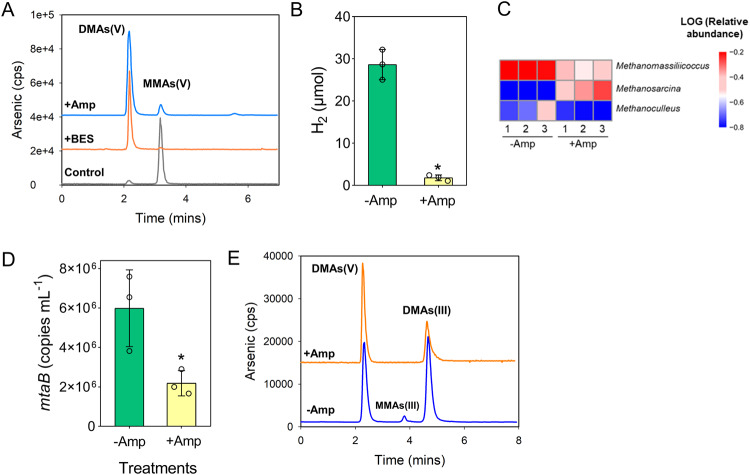
Methanogens and anaerobic bacteria synergistically demethylate DMAs(V). **A** Effect of BES (5 mM) or ampicillin (Amp, 100 mg l^−1^) on DMAs(V) demethylation by the methanol enrichment culture. Effect of Amp addition on (**B**) H_2_ production, (**C**) the composition of methanogens, (**D**) the absolute abundance of *Methanomassiliicoccus* specific *mtaB*, and (**E**) reduction of DMAs(V) to DMAs(III). The methanol enrichment cultures were amended with 20 μM DMAs(V). Data in (**B**) and (**D**) are means ± SD (*n* = 3). Arsenic species in (**A**) were oxidized by H_2_O_2_ to their corresponding pentavalent forms and separated via an anion-exchange column. Arsenic species in (**D**) were preserved with DDDC and separated via a C18 column. * in (**B**) and (**D**) indicates significant difference at *p* < 0.05 between the control and +Amp treatments (Tukey’s test).

Because *Methanomassiliicoccus* strains are obligate H_2_-dependent methylotrophic methanogens [38] and H_2_ is produced by some anaerobic bacteria [40], we examined whether bacteria play a role in DMAs(V) demethylation. As expected, the addition of BES, a specific inhibitor for methanogenic methyl-coenzyme M reductase, almost completely inhibited demethylation of DMAs(V) in the methanol enrichment culture (Fig. 5A). The addition of ampicillin, an antibiotic inhibiting most bacteria, produced a strong inhibiting effect on core genera of bacteria in the enrichment culture, including *Clostridium* sensu stricto 16, *Hydrogenispora*, *Clostridium* sensu stricto 13, and *Alkalibaculum* (Fig. S6). Ampicillin also inhibited DMAs(V) demethylation in the enrichment culture substantially (Fig. 5A). The addition of ampicillin decreased the concentration of H_2_ produced in the headspace of the enrichment culture by 93.7% (Fig. 5B). Sequencing of 16S rRNA in the enrichment culture showed that the addition of ampicillin decreased the abundance of *Methanomassiliicoccus* but increased the abundance of *Methanosarcina* (Fig. 5C). The abundance of hydrogenotrophic *Methanoculleus* was also decreased slightly by ampicillin, although its abundance was very low due to the use of methanol as the substrate to enrich methylotrophic methanogens. Quantification of the *mtaB* gene specific to *Methanomassiliicoccus* further confirmed the suppressive effect of ampicillin (Fig. 5D). The effect of ampicillin on the abundance of *Methanomassiliicoccus* in the enrichment culture was likely to be indirect, because it did not affect methane production by *Methanomassiliicoccus luminyensis* CZDD1 in pure culture during a time course of 48 days (Fig. S7).

In addition to producing H_2_ for methanogens, bacteria could also contribute to the reduction of DMAs(V) to DMAs(III), thus enhancing DMAs(V) demethylation. Indeed, the addition of ampicillin decreased the reduction of DMAs(V) to DMAs(III) in the methanol enrichment culture after incubation for 14 days (Fig. 5E). These results support the notion that the presence of some bacteria enhances DMAs(V) demethylation driven by some methylotrophic methanogens, such as those in the *Methanomassiliicoccus* genus, likely through enhancing their growth and the reduction of DMAs(V) to DMAs(III).

### Isolation of bacteria capable of H_2_ production and DMAs(V) reduction

To further examine the role of bacteria in DMAs(V) demethylation, we isolated three bacteria, *Clostridium malenominatum* CZB5, *Clostridium subterminale* CZB11, and *Tissierella carlieri* CZB10, from the methanol enrichment culture (Fig. S8). All three bacterial strains produced H_2_ (Fig. S9). Among the 145 bacterial MAGs constructed from metagenomic analysis of the methanol enrichment culture, 72 MAGs harbored Fe-Fe or Ni-Fe hydrogenase coding genes likely involved in H_2_ production (Fig. S3). All three bacterial strains, especially *Clostridium malenominatum* CZB5 and *Tissierella carlieri* CZB10, were able to reduce DMAs(V) to DMAs(III) (Fig. S10). For comparison, the ability to reduce DMAs(V) was relatively weak for *Methanosarcina mazei* CZ1 and negligible for *Methanomassiliicoccus luminyensis* CZDD1 (Fig. S10). Furthermore, all three bacterial strains could not demethylate DMAs(V) or DMAs(III) (data not shown).

### Synergistic effect between *Methanomassiliicoccus luminyensis* CZDD1 and *Clostridium malenominatum* CZB5 on DMAs(V) demethylation

**Fig. 6:**
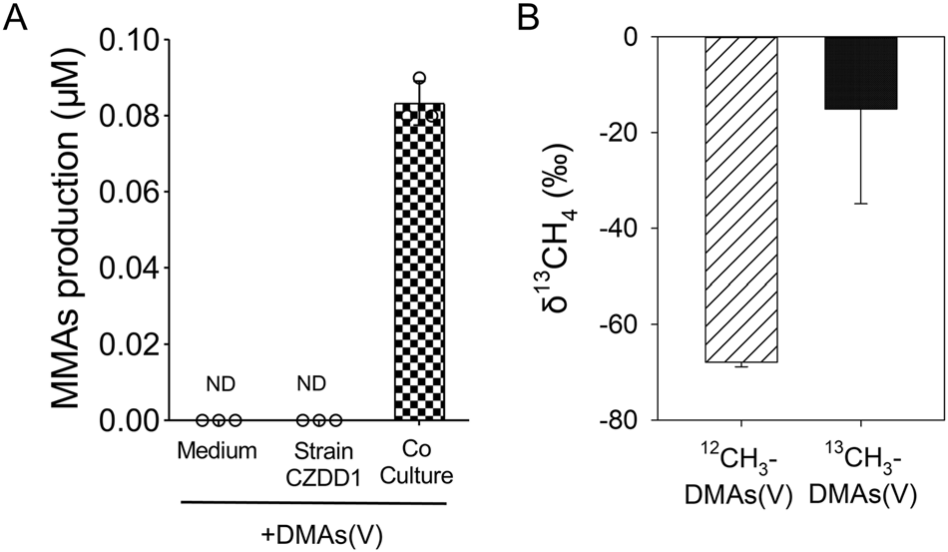
Demethylation of DMAs(V) by *Methanomassiliicoccus-Clostridium* coculture. **A** Production of MMAs in *Methanomassiliicoccus-Clostridium* coculture amended with 5 μM DMAs(V). **B** δ^13^C in CH_4_ produced by *Methanomassiliicoccus-Clostridium* coculture amended with 5 μM ^12^C-DMAs(V) or ^13^C-DMAs(V). Data are means ± SD (*n* = 3).

We tested whether coculture of *Methanomassiliicoccus luminyensis* CZDD1 with *Clostridium malenominatum* CZB5 could enable the former to demethylate DMAs(V). In the absence of exogenous H_2_, the coculture produced methane, reaching a peak on day 14 (Fig. S11A), suggesting that *Methanomassiliicoccus luminyensis* CZDD1 could utilize H_2_ produced by *Clostridium malenominatum* CZB5. Compared with the monoculture of *Methanomassiliicoccus luminyensis* CZDD1 supplemented with H_2_, *Methanomassiliicoccus luminyensis* CZDD1 grew much faster (measured by methane production) when cocultured with *Clostridium malenominatum* CZB5 (Fig. S11B). In contrast to monoculture of *Methanomassiliicoccus luminyensis* CZDD1, which did not demethylate DMAs(V), the coculture produced 0.08 μM MMAs from DMAs(V) demethylation after 30-day incubation (Fig. 6A). To confirm that the methyl group from DMAs(V) was converted to CH_4_, ^13^C-DMAs(V) was supplemented to the coculture. Compared with the control of adding non-label ^12^C-DMAs(V), methane produced by the coculture supplemented with^13^C-DMAs(V) was significantly enriched with ^13^C (Fig. 6B), indicating that ^13^C-DMAs(V) was demethylated to produce ^13^CH_4_.

### Augmentation of *Methanomassiliicoccus luminyensis* CZDD1 accelerates DMAs demethylation in paddy soil, decreases DMAs accumulation in rice and the incidence of straighthead disease

We tested whether the addition of *Methanomassiliicoccus luminyensis* CZDD1, *Clostridium malenominatum* CZB5, or the coculture of the two strains could accelerate the demethylation of DMAs produced by soil. TC paddy soil with a high As methylation potential [41] was preincubated under anoxic conditions for 4 days to allow the production of DMAs, to which inoculants of CZDD1, CZB5 or the coculture of the two strains were added. In the control, DMAs in the soil solution disappeared gradually during the following 15 days (Fig. S12). Augmentation of *Methanomassiliicoccus luminyensis* CZDD1 accelerated the disappearance of DMAs with concurrent increases in the concentrations of MMAs and iAs, whereas augmentation of *Clostridium malenominatum* CZB5 had no such effect. Augmentation of the coculture of *Methanomassiliicoccus*-*Clostridium* accelerated the disappearance of DMAs slightly faster than that of the *Methanomassiliicoccus luminyensis* CZDD1 monoculture (Fig. S12).

**Fig. 7:**
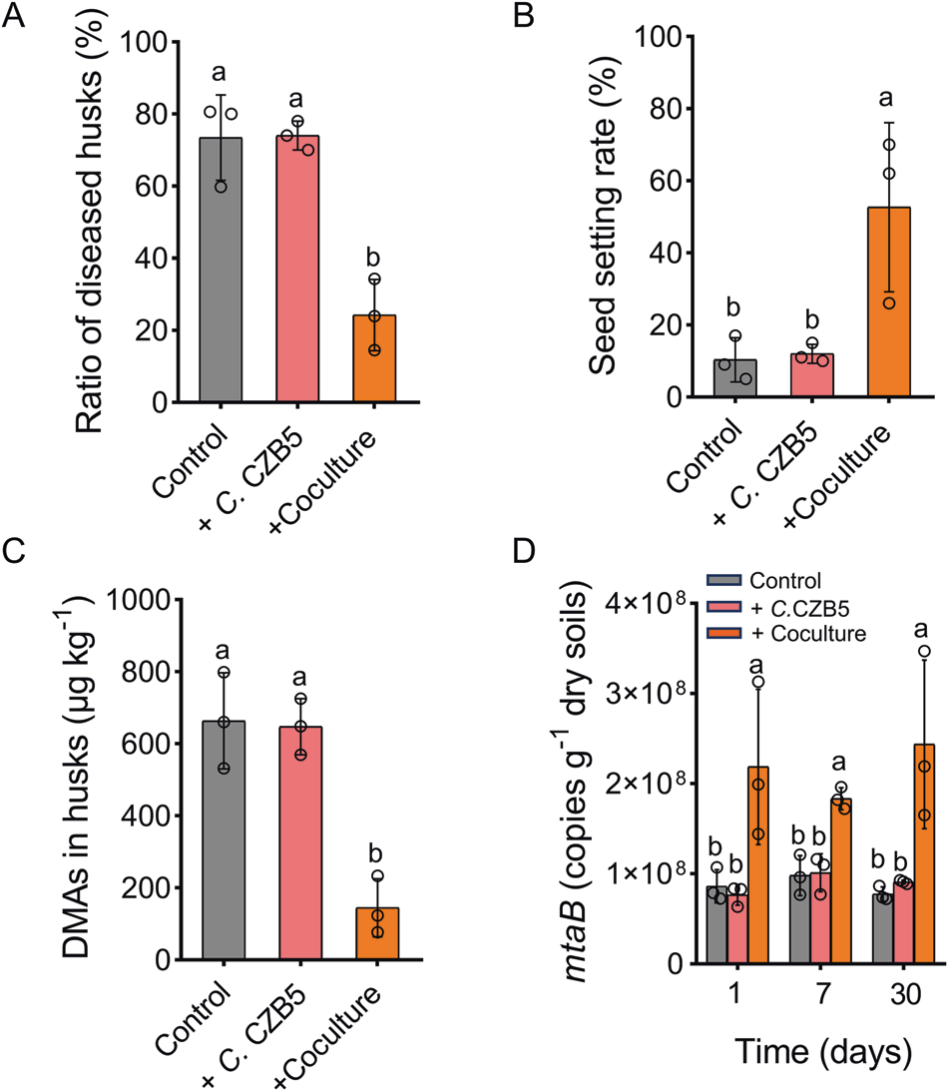
Addition of the *Methanomassiliicoccus*-*Clostridium* coculture decreased DMAs accumulation in rice husks and alleviated the incidence of straighthead disease of rice plants in a soil pot experiment. The effects of coculture and monoculture of *Clostridium malenominatum* CZB5 addition on (**A**) the percentage of rice grain with straighthead symptoms, (**B**) seed setting rate, (**C**) DMAs concentration in rice husks, and (**D**) the absolute abundance of *Methanomassiliicoccus* specific *mtaB*. Data are means ± SD (*n* = 3). Different letters indicate significant difference at *p* < 0.05 between treatments (Tukey’s test).

We grew rice in a pot experiment using TC paddy soil. At the late tillering stage, 150 ml of the *Clostridium malenominatum* CZB5 monoculture or the *Methanomassiliicoccus*-*Clostridium* coculture were added to the soil, with addition of uninoculated medium as the control. At the rice maturity stage, 73.5% of the rice grains from the control group showed distorted husks, a symptom characteristic of the straighthead disease. The augmentation of the *Methanomassiliicoccus*-*Clostridium* coculture decreased the percentage of rice grains with distorted husks to 24.3% (Fig. 7A). Meanwhile, the addition of coculture increased the seed setting rate from 10.3 to 52.7% (Fig. 7B) and decreased the concentration of DMAs in rice husks by 78.2% (Fig. 7C). In contrast, the addition of the *Clostridium malenominatum* CZB5 monoculture had no significant effect. Quantification of *mtaB* in the soil collected between 1–30 days after inoculation showed 1.9–3.1 fold higher gene copies in the treatment augmented with the *Methanomassiliicoccus*-*Clostridium* coculture than the control or the treatment with the *Clostridium malenominatum* CZB5 monoculture (Fig. 7D). These results indicate that augmentation of the *Methanomassiliicoccus*-*Clostridium* coculture decreased DMAs accumulation by rice plants and alleviated the straighthead disease, and the effect can be attributed to *Methanomassiliicoccus*.

### Variation in the DMAs(V) demethylation activity among paddy and upland soils

**Fig. 8:**
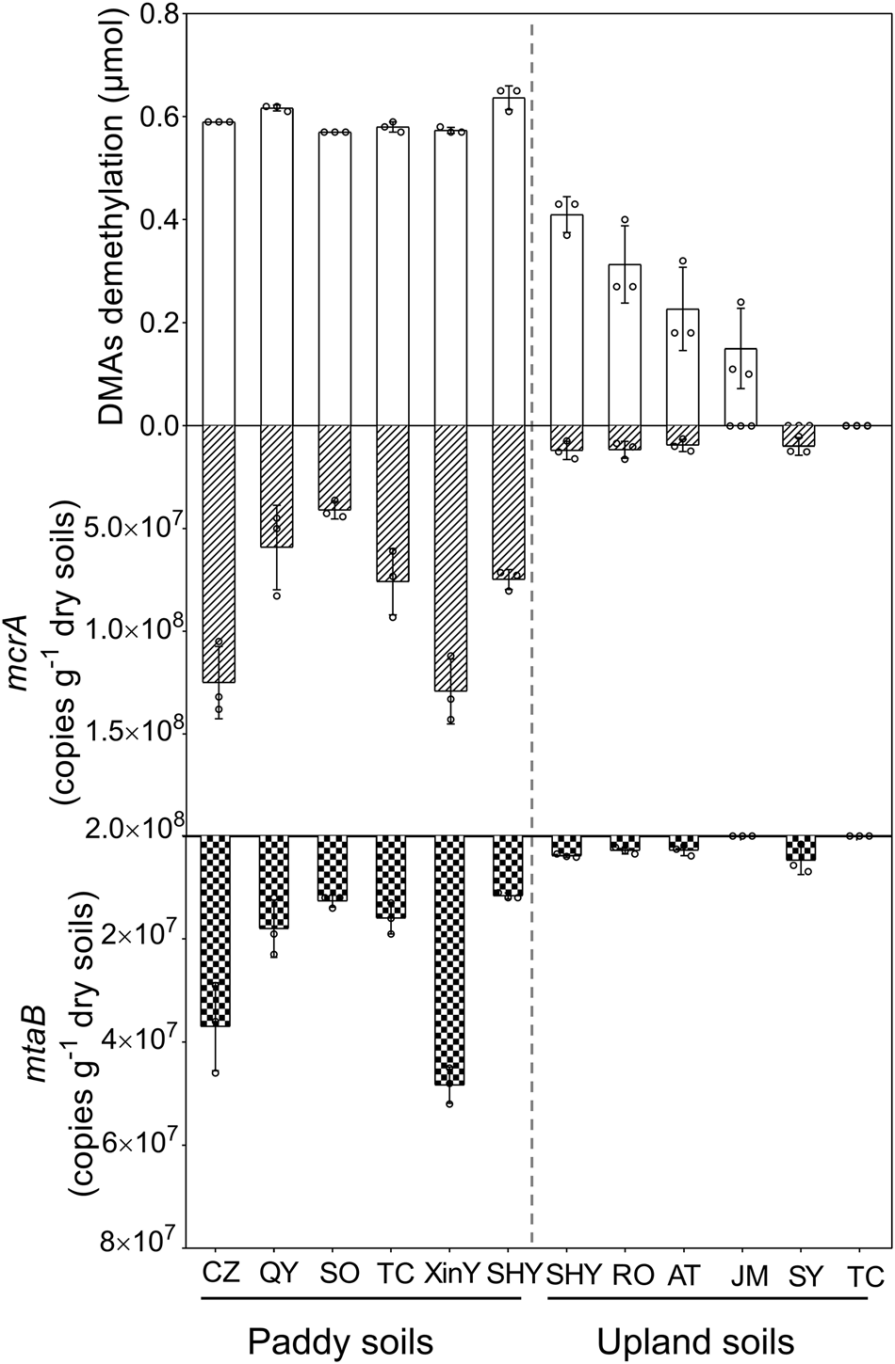
Paddy soils showed higher rates of DMAs(V) demethylation than upland soils. Differences between paddy and upland soils in the demethylation rate of DMAs(V), the absolute abundance of methanogen *mcrA* gene and *Methanomassiliicoccus* specific *mtaB* gene. Data are means ± SD (*n* = 3). Soil information is given in Table S3.

Long-term cultivation of paddy rice is likely to increase the population of methanogens in the soil [42], possibly leading to higher activity of DMAs(V) demethylation. We selected six paddy soils and six upland soils in an incubation experiment to measure demethylation of exogenous DMAs(V). After flooded incubation for 14 days, the exogenously added DMAs(V) (0.6 μmol kg^−1^ soil) was nearly completely demethylated in all six paddy soils, whereas substantial proportions (40–100%) of the added DMAs(V) was not demethylated in the six upland soils (Fig. 8). The abundances of both *mcrA* and *mtaB* genes were higher in the paddy soils than in the upland soil, with 3.4–2414.4- and 2.4–3247.8-fold differences between the two soil groups, respectively (Fig. 8). There were significant correlations between the percentage of DMAs(V) demethylation and the copy number of *mcrA* or *mtaB* when the data from all soils were combined, but there were no significant correlations in the separate groups of paddy or upland soils (Fig. S13).

## Discussion

We have previously shown that methanogens are likely involved in the demethylation of DMAs in anoxic paddy soils, based on the evidence from experiments using methanogenesis inhibitor and ^13^C-labeled DMAs [6]. However, the identity of methanogens responsible for DMAs demethylation was unknown. Consistent with our previous study [6], we found that BES completely inhibited DMAs demethylation. We further show that methylotrophic, rather than hydrogenotrophic and aceticlastic, methanogens are likely responsible for DMAs demethylation (Fig. 1). This is not surprising as methylotrophic methanogens can transfer methyl group from methylated compounds to cognate corrinoid protein and to coenzyme M, forming methyl-CoM that is subsequently reduced to methane [43, 44]. Other methylated compounds, such as methanol, methylated amines, methylated sulfide, dimethylselenide and methoxylated aromatic compounds, can also serve as substrates for methylotrophic methanogens [43, 45, 46].

*Methanomassiliicoccus* and *Methanosarcina* were the dominant genera (Fig. 1) in the methanol enrichment culture. Eight MAGs of *Methanomassiliicoccus* were also assembled from metagenomic analysis of the methanol enrichment culture. From the enrichment culture, we isolated two methylotrophic strains, *Methanomassiliicoccus luminyensis* CZDD1 and *Methanosarcina mazei* CZ1. *Methanomassiliicoccus luminyensis* CZDD1 is the first methanogen isolate in the order of Methanomassiliicoccales from paddy soils. Previously, *Methanomassiliicoccus luminyensis* B10 was isolated from human feces [38]. Strains of *Methanosarcina mazei* have been isolated from paddy soils [47] and wetland before [48]. Both *Methanomassiliicoccus luminyensis* CZDD1 and *Methanosarcina mazei* CZ1 are able to produce methane from methanol, although H_2_ is also required as the electron doner for the former as it lacks the Wood–Ljungdahl pathway [49, 50]. *Methanomassiliicoccus luminyensis* is known to be a H_2_-dependent methylotrophic methanogen, which uses H_2_ to reduce the methyl group to produce methane [50, 51]. We found that the pure culture of neither *Methanomassiliicoccus luminyensis* CZDD1 nor *Methanosarcina mazei* CZ1 could utilize DMAs(V), but the former could demethylate DMAs(III) to some extent (Fig. 6). When added to the methanol enrichment culture, *Methanomassiliicoccus luminyensis* CZDD1 greatly enhanced DMAs(V) demethylation, whereas *Methanosarcina mazei* CZ1 suppressed DMAs(V) demethylation likely by competing with the former (Fig. 3). Our study also establishes DMAs(III), not DMAs(V), as the substrate for demethylation (Fig. 4). This finding is similar to MMAs(III), rather than MMAs(V), being the substrate for bacterial demethylation [25, 39]. These findings suggest that the bond between the methyl group and trivalent As(III) is weaker and more prone to demethylation than that with pentavalent As(V). Although *Methanomassiliicoccus luminyensis* CZDD1 could not reduce DMAs(V), it could demethylate DMAs(III) that is reduced from DMAs(V) by other microorganisms. The ability to reduce DMAs(V) to DMAs(III) appears to be common among anaerobic bacteria [14]. Indeed, three anaerobic bacteria isolated from the methanol enrichment culture could reduce DMAs(V) to DMAs(III) (Fig. S10), and the coculture of *Methanomassiliicoccus luminyensis* CZDD1 with *Clostridium malenominatum* CZB5 enables the demethylation of DMAs(V) by the former. Moreover, the addition of ampicillin to the methanol enrichment culture suppressed bacteria-mediated reduction of DMAs(V), resulting in an inhibition of DMAs(V) demethylation driven by methylotrophic methanogens (Fig. 5). Compared with the enrichment culture, demethylation of DMAs(III) by *Methanomassiliicoccus luminyensis* CZDD1 or of DMAs(V) by the coculture of *Methanomassiliicoccus luminyensis* CZDD1 and *Clostridium malenominatum* CZB5 was relatively slow, suggesting the presence of other methanogens with a greater demethylation ability in the enrichment culture. Another possible explanation is that the concentration of methanol in the enrichment culture medium was three orders of magnitude higher than that of DMAs, which could competitively inhibit the uptake and utilization of DMAs by *Methanomassiliicoccus luminyensis* CZDD1. Competitive inhibition was observed between dimethylsulfide and dimethylselenide for demethylation by methylotrophic methanogens [46].

Apart from reduction of DMAs(V), anaerobic bacteria could also produce hydrogen for the growth of some H_2_-dependent methanogens, such as *Methanomassiliicoccus* sp. Hydrogen is produced by the [FeFe] or [NiFe] hydrogenases in some anaerobic bacteria during carbohydrate fermentation [52, 53]. The interspecies transfer of H_2_ between bacteria and methanogens helps sustain the growth in syntrophic methanogenic communities [54–56]. In the present study, genes coding for [FeFe] or [NiFe] hydrogenases are widely present in the bacterial MAGs of the methanol enrichment culture (Fig. S3). Three bacterial isolates from the methanol enrichment culture are all capable of producing H_2_ and promoting methane production in a coculture with *Methanomassiliicoccus luminyensis* CZDD1 (Figs. S9 and S11). In addition, ampicillin suppressed the production of H_2_ in the methanol enrichment culture as well as the abundance of *Methanomassiliicoccus* sp. (Fig. 5). These results support a synergistical relationship between *Methanomassiliicoccus* sp. and some anaerobic bacteria, with bacteria providing H_2_ for growth of *Methanomassiliicoccus* sp. and reducing DMAs(V) prior to demethylation.

We further demonstrate that the addition of a coculture of *Methanomassiliicoccus luminyensis* CZDD1 and *Clostridium malenominatum* CZB5 to paddy soil decreased DMAs accumulation by rice plants and, as a result, alleviated straighthead disease (Fig. 7). This effect was produced by the methanogen as the addition of *Clostridium malenominatum* CZB5 alone had no such effect. These results suggest that DMAs demethylation in paddy soil can be enhanced by augmentation of *Methanomassiliicoccus*. In contrast, suppression of methanogenesis in paddy soil by BES was found to increase the severity of straighthead disease in rice [57].

In anoxic paddy soil, DMAs(V) can be demethylated completely to arsenite [6, 26]. In the present study, however, we found that *Methanomassiliicoccus* sp. only partially demethylates DMAs(III), producing MMAs(III) as the main product. Further demethylation of MMAs(III) requires other microorganisms. It has been shown that MMAs(III) can be further demethylated to arsenite by bacteria harboring the C−As lyase enzyme ArsI [25]. In anoxic paddy soil, some denitrifying bacteria possessing ArsI are able to demethylate MMAs(III) to arsenite [25]. Thus, complete demethylation of DMAs(V) in paddy soil requires concerted actions of multiple microorganisms, from bacteria mediating DMAs(V) reduction, methylotrophic methanogens such as *Methanomassiliicoccus* sp. demethylating DMAs(III), and bacteria possessing ArsI for further demethylation of MMAs(III). Demethylation of DMAs(III) by methylotrophic methanogens appears to be the rate-limiting step, because MMAs(III) does not persist in anoxic paddy soil [6, 25]. Compared with paddy soils, upland soils have a smaller population of methylotrophic methanogens and also demethylate DMAs(V) at a slower rate (Fig. 8). In fact, rice straighthead disease is often prevalent when upland soils are converted to paddy fields; the disease rarely occurs in old paddy soils [22, 23]. A possible explanation is that newly converted paddy soils have low populations of methylotrophic methanogens to demethylate DMAs.

In conclusion, we have identified *Methanomassiliicoccus luminyensis* CZDD1 from paddy soil as a methanogen capable of demethylating DMAs(III). Anaerobic bacteria also play a role by reducing DMAs(V) to DMAs(III) and generating H_2_ for the growth of H_2_-dependent methylotrophic methanogens. Augmentation of *Methanomassiliicoccus*–*Clostridium* coculture enhanced DMAs demethylation in paddy soil, which could provide an effective strategy for controlling rice straighthead disease caused by DMAs accumulation.

### Supplementary information

Supplementary Tables and Figures

## Data Availability

The raw sequences of 16S rRNA have been deposited in the NCBI SRA database under the accession numbers PRJNA909841 for bacteria and PRJNA909967 and PRJNA1003412 for archaea in enrichment cultures. The MAG genomic sequences are deposited in the NCBI Genome database under the BioProject ID PRJNA1004092. The sequencing data of 16S rRNA of methanogenic isolates are under the NCBI GenBank accession OQ554539 and OQ566822, the accession number for bacterial isolates are OQ548091, OQ566864 and OQ560997.
