# Supplementary material for: Methylotrophic methanogens and bacteria synergistically demethylate dimethylarsenate in paddy soil and alleviate rice straighthead disease
Source: ISME J. 2023 Aug 21;17(11):1851–61. doi: 10.1038/s41396-023-01498-7 (PMC10579292; doi:10.1038/s41396-023-01498-7)

Methylotrophic methanogens and bacteria synergistically demethylate dimethylarsenate in paddy soil and alleviate rice straighthead disease

Chuan Chen, Lingyan Li, Yanfen Wang, Xiuzhu Dong and Fang-Jie Zhao

Supplementary Tables and Figures

Table S1. Compositions of the methanogenic medium used in the present study.

Name	Compose
Base medium	Tryptone 0.25 g L^{-1}, Yeast Extract 0.5 g L^{-1}, Soya peptone 0.25 g L^{-1}, $NaHCO_3$ 3.92 g L^{-1}, NaCl 2.92 g L^{-1}, L-Cysteine 0.25 g L^{-1}, Resazurin 0.01%, Methanol 20 mM.
Mineral solution (MSV)	NH_4Cl 6 g L^{-1}, NaCl 6 g L^{-1}, $CaCl_2$ •$2H_2O$ 0.2 g L^{-1}, $MgCl_2$ •$6H_2O$ 2 g L^{-1}.
Solution I	0.2 M KH_2PO_4 (20 mL L^{-1}).
Solution II	0.2 M Na_2HPO_4 (47 mL L^{-1}).
Trace element solution	$FeCl_2$•$4H_2O$ 2 g L^{-1}, $MnCl_2$•$4H_2O$ 0.05 g L^{-1}, $(NH_4)_6Mo_7O_{24}$ •$4H_2O$ 0.05 g L^{-1}, $ZnCl_2$ 0.05 g L^{-1}, $CuCl_2$ •$2H_2O$ 0.03 g L^{-1}, $AlCl_3$ 0.05 g L^{-1}, $CoCl_2$•$6H_2O$ 0.2 g L^{-1}, Na_2SeO_3 2mg L^{-1}, Na_2WO_4 4 mg L^{-1}, NaOH 0.4 g L^{-1}, 0.4 H_3BO_3 saturated solution (1 mL L^{-1}).
Vitamin solution	Biotin 0.002 g L^{-1}, Folic acid 0.002 g L^{-1}, Pyridoxine-HCl 0.01 g L^{-1}, Riboflavin 0.005 g L^{-1}, Thiamine-HCl 0.005 g L^{-1}, Niacin 0.005 g L^{-1}, Cyanocobalamin 0.005 g L^{-1}, *p*-aminobenzoic acid, 0.005 g L^{-1}, Pantothenic acid 0.005 g L^{-1}.
MTV complex solution (125 μL/5 mL)	$V_{\text{Mineral solution}}$: $V_{\text{Trace element solution}}$: $V_{\text{vitamin solution}}$=96: 2: 2

Table S2. Primers used in the present study.

Number	Names	Sequences
P1	mlas	GGTGGTGTMGGDTTCACMCARTA
P2	*mcrA*-rev	CGTTCATBGCGTAGTTVGGRTAGT
P3	*mtaB*-F	GCTGATGCAGAAGTACCRYGA
P4	*mtaB*-R	GTADCCRATRCCGAACAGCCA
P5	515F	GTGCCAGCMGCCGCGG
P6	907R	CCGTCAATTCMTTTRAGTTT
P7	Arch519F	CAGCCGCCGCGGTAA
P8	Arch915R	GTGCTCCCCCGCCAATTCCT
P9	met86F	GCTCAGTAACACGTGG
P10	met1340R	CGGTGTGTGCAAGGAG
P11	27F	AGAGTTTGATCMTGGCTCAG
P12	1492R	TACGGYTACCTTGTTACGACTT

Table S3. Locations and properties of the paddy and upland soils used in the present study.

Type	Code	pH	Soil organic matter ($g\ kg^{-1}$)	Location
Paddy Soils	CZ	6.7	30.8	Chenzhou, Hunan province, China
	QY	6.0	21.9	Qiyang, Hunan province, China
	SO	6.8	21.4	Sonargaon, Bangladesh
	TC	5.8	9.9	Tancheng, Shandong province, China
	XinY	6.3	28.5	Xinyang, Henan province, China
	SHY	5.9	31.4	Shuyang, Jiangsu province, China
Upland Soils	SHY	7.0	38.9	Shuyang, Jiangsu province, China
	RO	6.5	30.5	Rothamsted, UK
	AT	4.8	22.7	Athens, GA, USA
	JM	4.7	11.1	Jinmen, Hubei province, China
	SY	7.1	30.5	Siyang, Jiangsu province, China
	TC	5.1	10.2	Tancheng, Shandong province, China

Table S4. Mass balance and distribution of arsenic species in the soil solution and solid phases, and volatile arsenic in the headspace of the methanol enrichment cultures amended with or without DMAs (80 μM x 5 mL = 0.4 μmol per bottle) at the end of the incubation experiment. Data are means ± SE (*n* = 3).

Treatments	Solution Phase (μmol)		Solid Phase (μmol)		Volatile As (μmol)	Demethylated As from DMAs (μmol)	Recovery (%)
	MMAs	iAs	MMAs	iAs			
+Methanol +DMAs	0.162 ± 0.008	0.169 ± 0.002	0.086 ± 0.006	0.164 ± 0.015	0.00016 ± 0.00006	0.394	98.5
+Methanol	ND	0.098 ± 0.016	ND	0.089 ± 0.003	/ND	/	

ND, not detectable.

Figure S1. Design of a pair of primers specific for *Methanomassiliicoccus* based on *mtaB* gene. **A** Alignment of *mtaB* genes obtained from strains or MAGs of *Methanomassiliicoccus* and the two conserved regions selected for primer design. Agarose gel electrophoresis of *mtaB* genes amplified with **B** genome DNA extracted from strain *Methanomassiliicoccus luminyensis* CZDD1 or *luminyensis* B10 and *Methanosarcina maize* CZ1 and **C** total DNA of paddy soil and methanol enrichment culture as templates to verify the specificity of the primers. **D** Identification of fragment of *mtaB* genes via constructing a clone library using the designed primer.

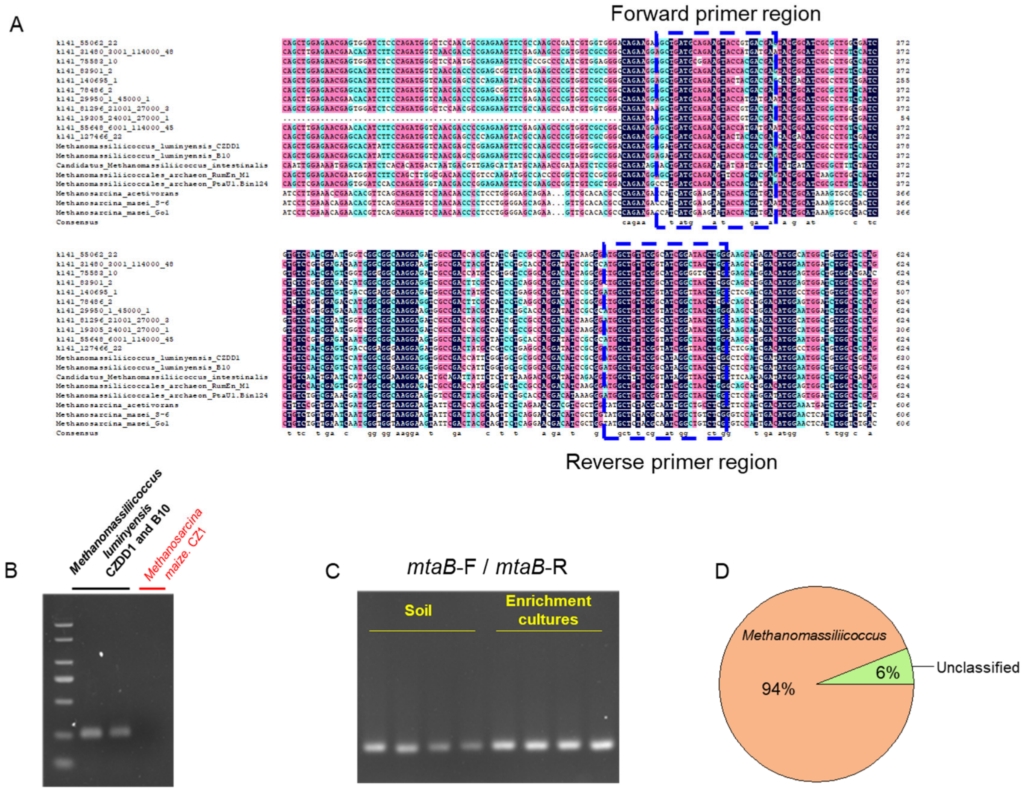

Figure S2. Production of inorganic arsenic (iAs) in different enrichment cultures of methanogens amended with 20 μM DMAs(V) during incubation.

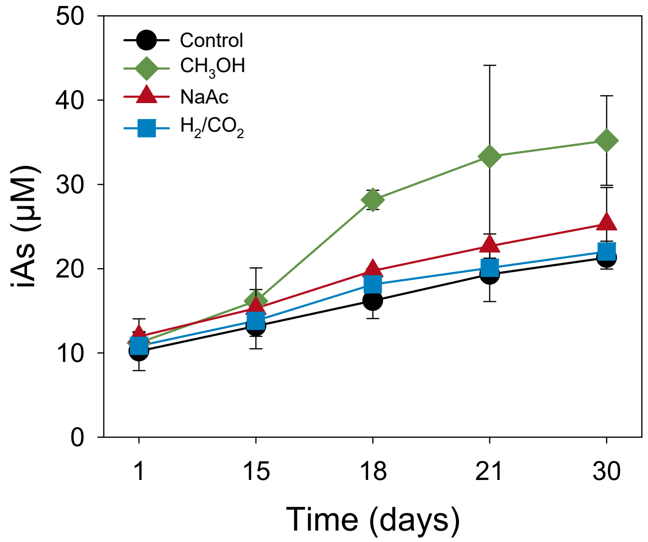

Figure S3. Bacterial and methanogenic metagenome-assembled genomes (MAGs) obtained via metagenomic analysis of the methanol enrichment culture. Yellow circles indicate bacterial MAGs harboring Fe-Fe or Ni-Fe hydrogenases.

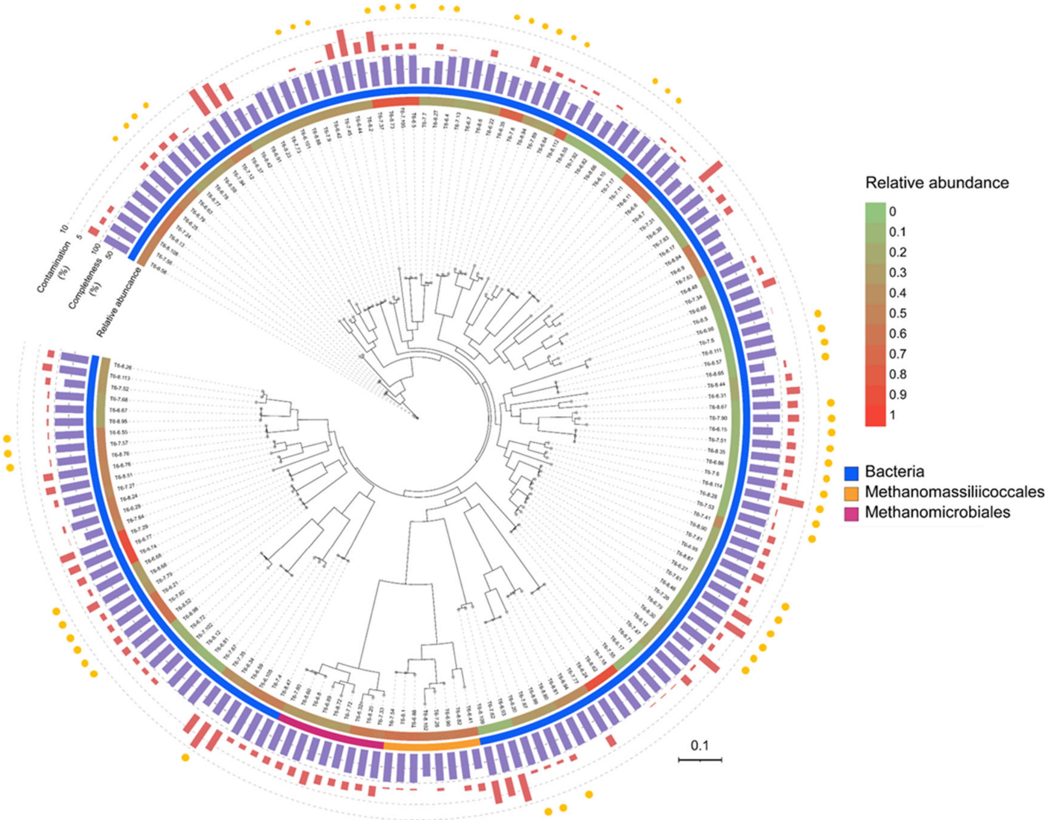

Figure S4. No demethylation of DMAs(V) (5 μM) by the mono-culture of *Methanomassiliicoccus luminyensis* CZDD1 or *Methanosarcina maize* CZ1.

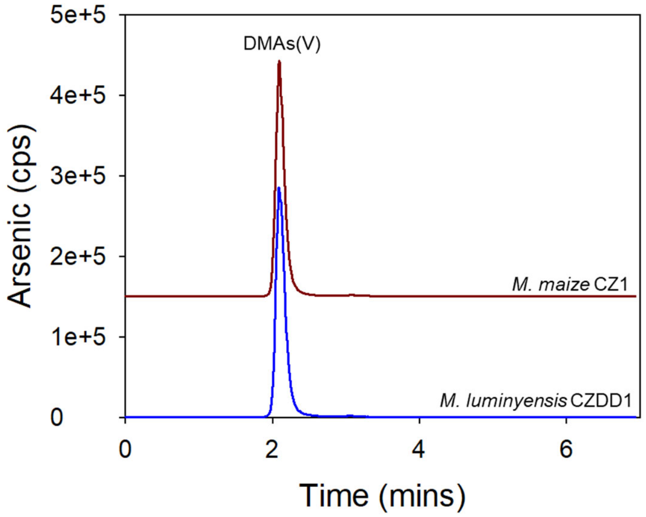

Figure S5. Effect of *Methanomassiliicoccus luminyensis* CZDD1 addition to the methanol enrichment culture on the relative abundance of core genera of methanogens. Data are means ± SD (n = 3).

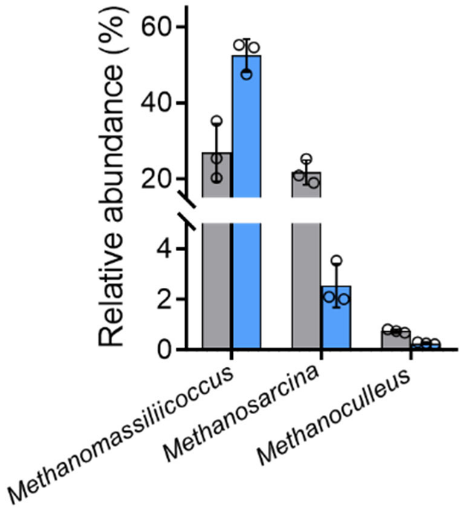

Figure S6. Effect of ampicillin addition to the methanol enrichment culture on the relative abundance of bacteria. Only those with an abundance of >0.1% are shown in the Figure. Data are means ± SD (n = 3).

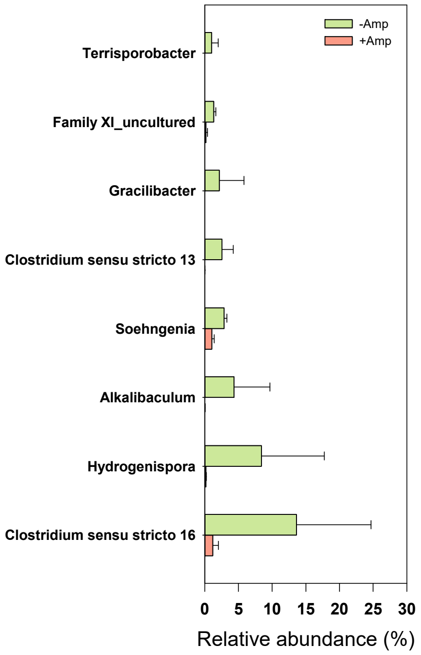

Figure S7. Effect of ampicillin addition on methane production by *Methanomassiliicoccus luminyensis* CZDD1. Data are means ± SD (n = 3).

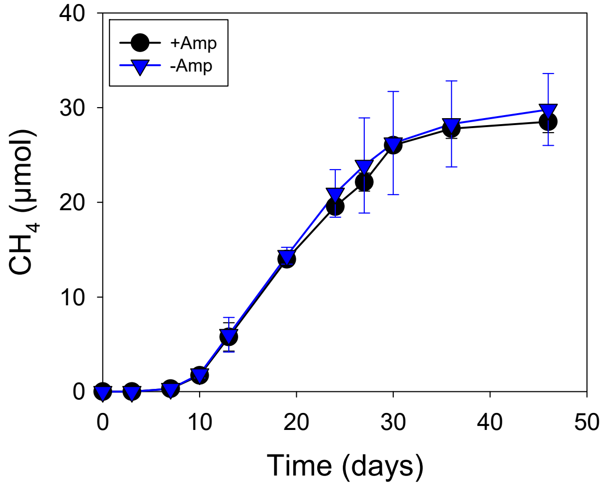

Figure S8. Phylogenetic analysis of bacteria isolated from the methanol enrichment culture of CZ paddy soil. Strains in bold are those isolated in the present study.

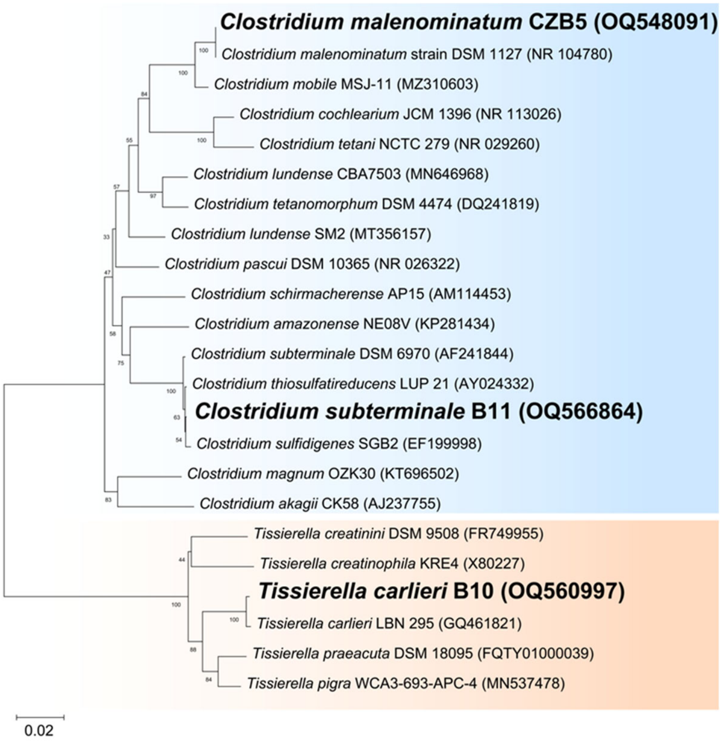

Figure S9. Hydrogen (H_2) production in the headspace of bacterial strains *Clostridium malenominatum* CZB5, *Tissierella carlieri* B10 and *Clostridium subterminale* CZB11. Data are means ± SD (n = 3).

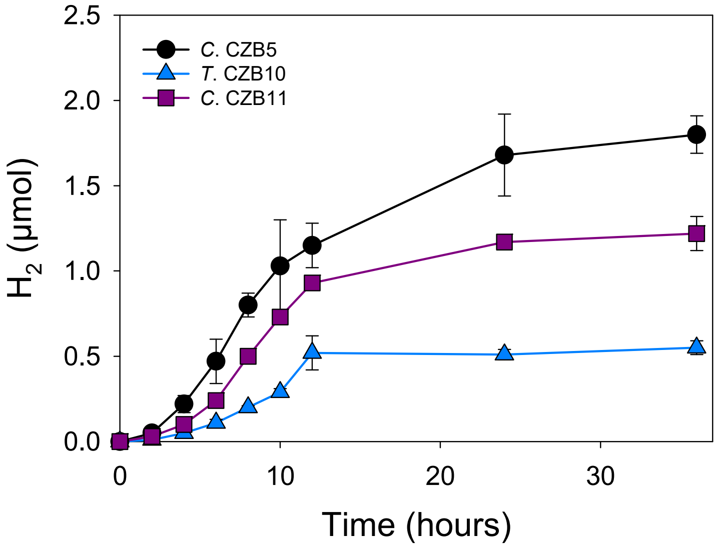

Figure S10. Reduction of DMAs(V) to DMAs(III) by bacterial strains (**A, B, C**) and methanogenic strains (**D, E**) isolated from the methanol enrichment culture of CZ paddy soil.

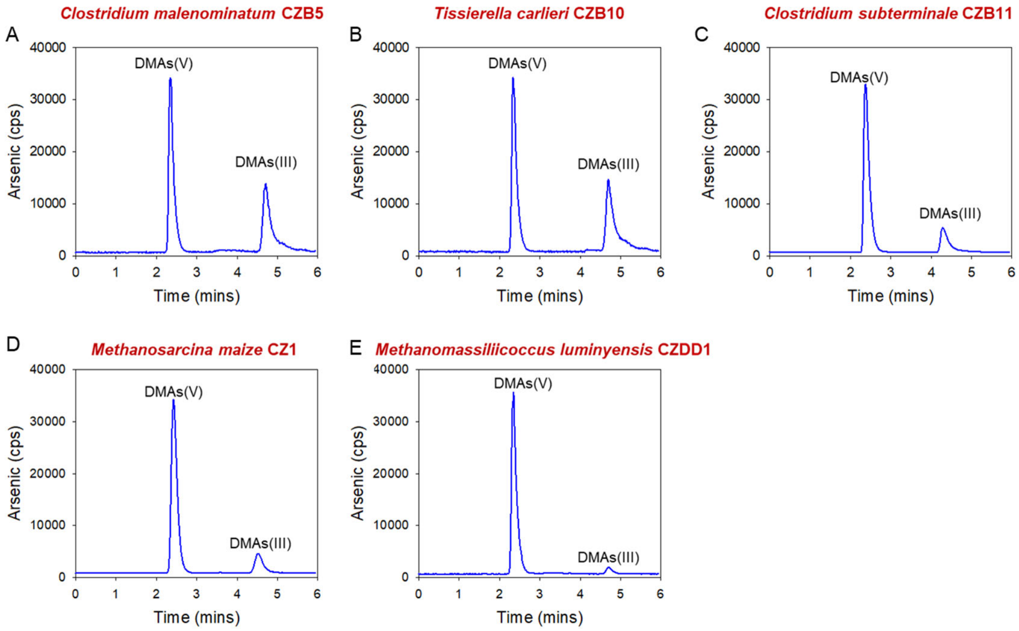

Figure S11. Methane production in the coculture of *Methanomassiliicoccus luminyensis* CZDD1 with *Clostridium malenominatum* CZB5, *Tissierella carlieri* B10 or *Clostridium subterminale* B11 (**A**), and in the coculture of *Methanomassiliicoccus luminyensis* CZDD1 with *Clostridium malenominatum* CZB5 compared with monoculture of *Methanomassiliicoccus luminyensis* CZDD1 supplemented with exogenous H_2 (0.1 MPa) (**B**). Data are means ± SD (n = 3).

A

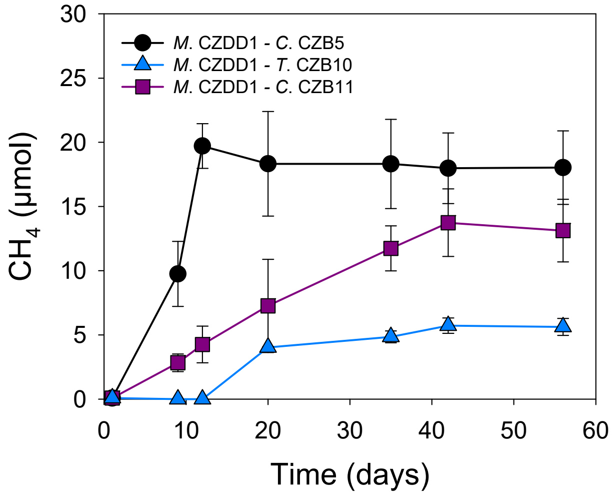

B

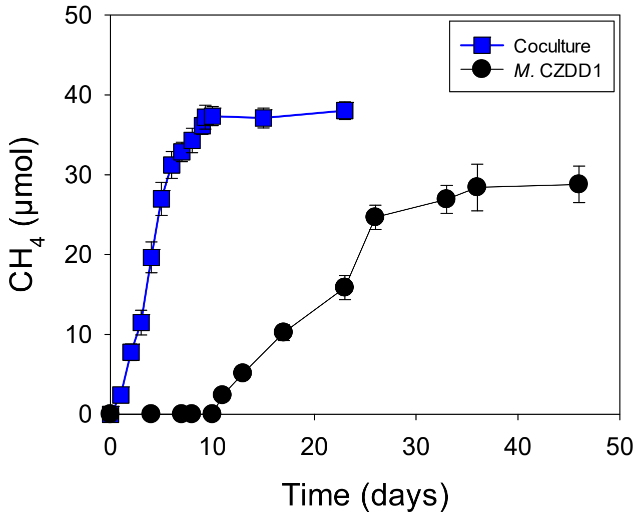

Figure S12. Effects of the addition of the monoculture of *Methanomassiliicoccus luminyensis* CZDD1 or *Clostridium malenominatum* CZB5, or the *Methanomassiliicoccus*-*Clostridium* coculture on DMAs demethylation in TC paddy soils. Changes in the concentrations of **A** DMAs, **B** MMAs and **C** iAs in soil porewater. Data are means ± SD (n = 3).

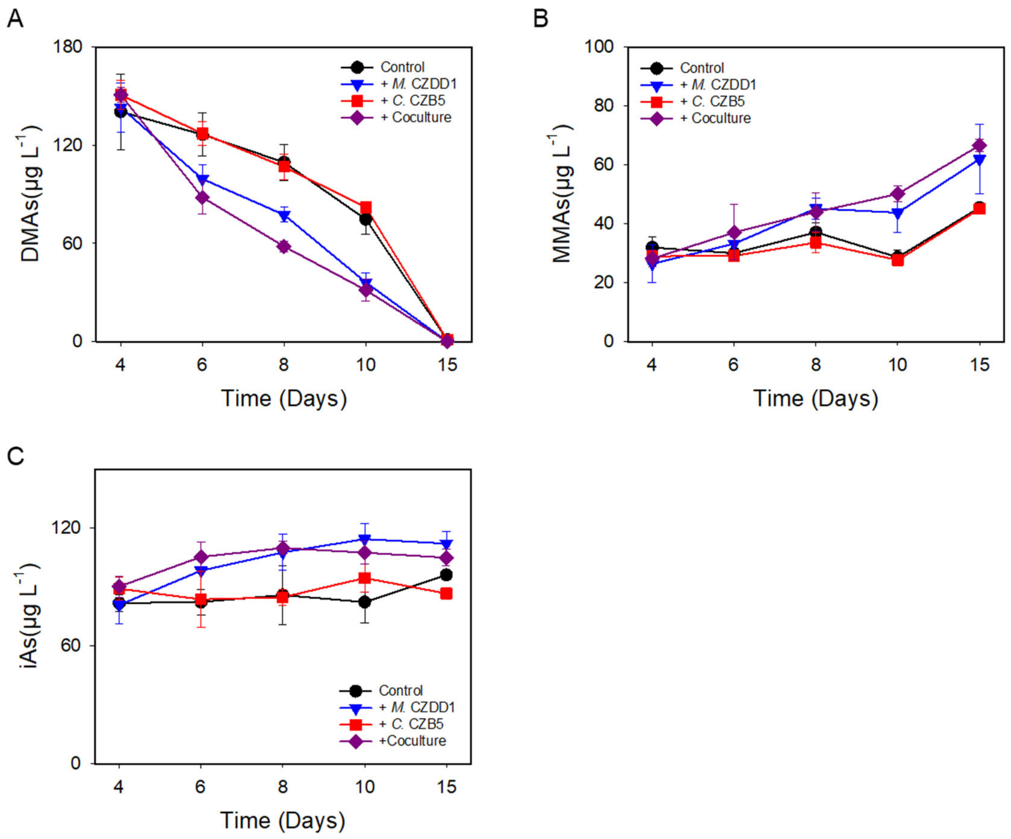

Figure S13. Correlations between the percentage of DMAs(V) demethylation and the copy number of *mcrA* (**A, C, E**) or *mtaB* (**B, D, F**) in all paddy and upland soils (**A, B**), or within the paddy soils (**C, D**) or upland soils (**E, F**). Each symbol represents one replicate of a soil.

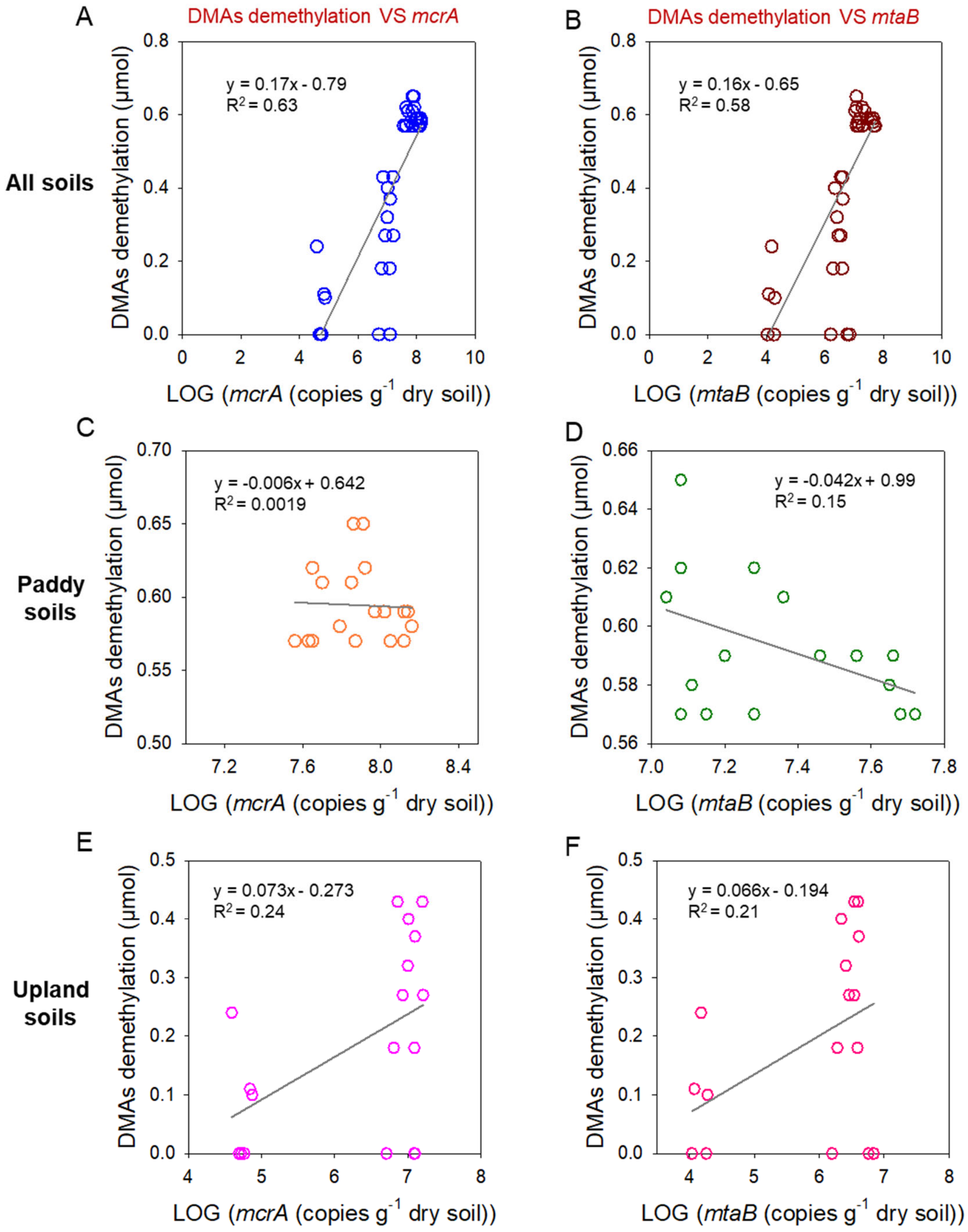

Supplement: Supplementary file 1 — Supplementary Tables and Figures [file 41396_2023_1498_MOESM1_ESM.pdf]
